# Profiling triple-negative breast cancer-specific super-enhancers identifies high-risk mesenchymal development subtype and BETi-Targetable vulnerabilities

**DOI:** 10.1186/s12943-025-02342-6

**Published:** 2025-05-13

**Authors:** Qing-shan Chen, Rui-zhao Cai, Yan Wang, Ge-hao Liang, Kai-ming Zhang, Xiao-Yu Yang, Dong Yang, De-Chang Zhao, Xiao-Feng Zhu, Rong Deng, Jun Tang

**Affiliations:** 1 State Key Laboratory of Oncology in South China, Guangdong Provincial Clinical Research Center for Cancer, Sun Yat-sen University Cancer Center, Guangzhou, 510060 P. R. China; 2 Department of Breast Oncology, Sun Yat-sen University Cancer Center, Guangzhou, China

**Keywords:** Super-enhancers, Triple-negative breast cancer, Heterogeneity, Transcription factors

## Abstract

**Background:**

Super-enhancers (SEs) are critical regulators of tumorigenesis and represent promising targets for bromodomain and extra-terminal domain inhibitors (BETi). However, clinical studies across various solid tumors, including triple-negative breast cancer (TNBC), have demonstrated limited BETi efficacy. This study aims to investigate SE heterogeneity in TNBC and its influence on BETi effectiveness, with the goal of advancing BETi precision treatment strategies and enhancing therapeutic efficacy.

**Methods:**

We conducted a comprehensive analysis of H3K27ac ChIP-Seq data from TNBC cell lines and clinical samples, integrating multiple bulk RNA-Seq, scRNA-Seq, and stRNA-Seq datasets to characterize the SE landscape and heterogeneity in TNBC. Utilizing various bioinformatics algorithms, CERES scoring, and clinical prognostic data on transcription factors (TFs), we identified core transcriptional regulatory circuits (CRCs) composed of TNBC-specific SEs and master regulators, characterizing different TNBC subtypes. The biological significance of CRCs in these different TNBC subtypes and their influence on BETi sensitivity were evaluated using in vitro and in vivo models.

**Results:**

Our findings revealed a distinct SE landscape in TNBC compared to non-TNBC and normal breast epithelium, allowing TNBC to be classified into distinct subtypes based on TNBC-specific SEs. Importantly, we identified a high-risk mesenchymal development subtype, validated across cell lines and transcriptomic analyses, primarily driven by a CRC consisting of the master regulator VAX2 and a TNBC-specific SE. This SE-VAX2 CRC is essential for sustaining the malignant traits of this subtype and increasing its sensitivity to BETi.

**Conclusions:**

Our research clarifies the heterogeneity of SEs in TNBC and identifies a high-risk mesenchymal development subtype driven by the SE-VAX2 CRC. The subtype shows more sensitivity to BETi, supporting its precision application in TNBC.

**Supplementary Information:**

The online version contains supplementary material available at 10.1186/s12943-025-02342-6.

## Background

Breast cancer remains one of the most prevalent malignant tumors in women [1], as triple-negative breast cancer (TNBC) garners significant attention due to its aggressive nature and pronounced heterogeneity [2, 3]. TNBC can be subdivided into multiple subtypes based on driver gene mutations [4, 5], transcriptional expression patterns [6–8], and protein regulatory networks [9, 10], each exhibiting distinct biological characteristics and therapeutic potential. Despite progress in understanding TNBC heterogeneity, most studies have predominantly relied on clustering analyses of gene expression profiles, often failing to distinguish primary oncogenic drivers from secondary effectors. SEs are specialized cis-regulatory elements located in non-coding regions, formed by clusters of adjacent active enhancers [11]. These SEs recruit numerous transcriptional co-factors, such as p300 and BRD4, along with key transcription factors (TFs), ultimately leading to the dysregulation of downstream target genes when aberrantly activated [12]. Compared with conventional enhancers, SEs play a crucial role in initiating and sustaining malignancies [13]. Thus, SEs may serve as more precise driver markers for cancer subtypes, and analyzing SE heterogeneity could provide deeper insights into tumor biology.

SE heterogeneity has been documented across various tumor types [14], including neuroblastoma [15], medulloblastoma [16], acute myeloid leukemia [17], colorectal cancer [18], multiple myeloma [19], gastric cancer [20], and bladder cancer [21]. This heterogeneity categorizes tumors into subtypes with distinct biological functions, regulated by specific SEs and their associated TFs. Among these, TFs that preserve subtype-specific characteristics function as master regulators. For example, SE heterogeneity analysis has identified SOX10 as the master regulator of the RTK I subtype in glioblastoma [22], HLX as the master regulator of the Group 3 subtype in medulloblastoma, and LMX1A as the master regulator of the Group 4 subtype in medulloblastoma [16]. These findings highlight the potential of SE landscape analysis in elucidating tumor heterogeneity. Identifying subtype-specific master regulators not only deepens our understanding of tumor biology but also offers novel avenues for targeted therapies. However, a comprehensive analysis of SE heterogeneity and its associated master regulators in TNBC remains to be performed.

Given the critical role of SEs and master regulators in driving malignant processes, disrupting SE-dependent transcriptional activity represents a promising strategy for cancer treatment [23]. BRD4, a member of the bromodomain and extra-terminal domain (BET) family, is extensively recruited at SEs, making BET inhibitors (BETi) effective in disrupting SE function [24–26]. Although preclinical models of TNBC have demonstrated the efficacy of BETi [27–31], clinical trials in solid tumors, including TNBC, have yielded mixed results [32–36]. While some patients experienced partial responses, the overall response rate remained low, and unexpected toxicity further limited clinical efficacy, likely due to a lack of specificity for molecular subtypes [37]. These observations underscore the urgent need for a comprehensive analysis of TNBC SE heterogeneity and its associated transcriptional regulation to overcome these challenges. Therefore, our study aims to investigate SE heterogeneity in TNBC and its impact on BETi treatment, with the goal of identifying therapeutic targets and developing personalized treatment strategies for appropriate patients.

In this study, our analysis revealed a higher number of TNBC-specific SEs, which exhibited significant heterogeneity. These SEs allowed us to subdivide TNBC into distinct subtypes, with one consistently identified as the mesenchymal development subtype, characterized by higher malignancy, poorer prognosis, strong cellular interactions with cancer-associated fibroblasts (CAFs), and enrichment in an extracellular matrix (ECM)-associated tumor microenvironment (TME). To identify the key SEs and master regulators driving this subtype, we employed a comprehensive approach integrating CRCmapper, ARACNe, pySCENIC algorithms, CRISPR/CERES scores, clinical prognosis analysis, and TF pseudotime analysis. This identified a core regulatory circuit (CRC) driven by SE-regulated VAX2, with VAX2 emerging as the key master regulator of the mesenchymal development subtype. Further in vitro and in vivo studies demonstrated VAX2’s critical role in regulating the mesenchymal development subtype of TNBC and driving tumor progression. Additionally, the SE-TF cooperation within the CRC increased the susceptibility of associated TFs to perturbations, making the mesenchymal development subtype more responsive to BETi. Finally, to enhance clinical applicability, we developed a machine learning model based on the regulation of VAX2, incorporating a 27-gene signature to predict the mesenchymal development subtype of TNBC and guide precise treatment strategies.

## Methods

### H3K27ac ChIP-seq processing

The sources of all H3K27ac ChIP-seq data are detailed in Supplementary Table 1. ChIP-seq reads were first trimmed using Trim-Galore and subsequently aligned to the human reference genome (hg19/GRCh37) via Bowtie2. To improve data quality, duplicate and multi-mapping reads were removed using Sambamba. Peak calling was performed with the callpeak mode in MACS2. Input and corresponding immunoprecipitation datasets were normalized using the signal extraction scaling method and converted to bigWig tracks via the bamCompare tool in deepTools2. Quality control of the ChIP-seq datasets was rigorously performed, assessing mapping statistics, enrichment levels, and library complexity metrics, including the PCR bottlenecking coefficients (PBC1 and PBC2) and the non-redundant fraction (NRF). Additional quality metrics such as cross-correlation scores (NSC and RSC) and the fraction of reads in peaks (FRiP) were evaluated following ENCODE data standards (https://www.encodeproject.org/chip-seq/histone/). Only primary H3K27ac ChIP-seq datasets meeting the following criteria were included in the analysis: PBC1 ≥ 0.5, PBC2 ≥ 1, NRF ≥ 0.5, NSC ≥ 1.05, RSC ≥ 0.8, total mapped reads ≥ 10 million, and MACS2 peak count ≥ 10,000.

### H3K27ac ChIP-seq diffbind analysis

The DiffBind R package was used to identify differentially enriched ChIP-seq regions among TNBC, non-TNBC, and normal breast epithelial samples. The dba and dba.count functions in DiffBind were applied to identify shared peaks across all samples. A matrix of normalized read counts was then generated, from which the top 10,000 most variable sites were selected for further analysis. To visualize differences between TNBC, non-TNBC, and normal breast epithelial samples, an MDS plot was constructed.

### Identification of enhancer and SE

H3K27ac typical enhancers and SEs in each sample were identified using the ROSE algorithm (https://bitbucket.org/young_computation/rose/src/master/). Peaks located within 3 kb upstream or downstream of transcription start sites were filtered out, and those listed in the ENCODE blacklist were excluded from further analysis. The remaining peaks were processed using the default parameters of the ROSE algorithm. The H3K27ac signal over the consensus SEs was quantified using the bigWigAverageOverBed tool. To identify TNBC-specific SEs, we compared consistent SE regions and H3K27ac signals across TNBC, non-TNBC, and normal breast epithelial samples. The computeMatrix and plotHeatmap functions in deepTools were used to visualize the H3K27ac signal within a 10 kb region upstream and downstream of the TNBC-specific SEs.

### Assigning TNBC-specific SEs targets genes

To accurately identify target genes regulated by TNBC-specific SEs, Hi-C data from GSE167154 were obtained for the BT549 and HCC70 TNBC cell lines, as well as TNBC tissue samples. Hi-C data were used to analyze target genes that were spatially associated with SEs through direct chromatin interactions. Genes interacting with SEs via these chromatin contacts were identified as being directly regulated by the SEs. For SEs without directly interacting genes, genes within a 1 Mb region surrounding the SE were screened for significant correlation (Spearman’s *R* > 0.7) between mRNA expression and the SE H3K27ac signal. Genes meeting this correlation threshold were also considered SE-regulated. If no significant expression-H3K27ac correlation was found, the gene closest to the SE was designated as its target.

### BRCA cell lines and sample mRNA datasets

Gene expression data for BRCA cell lines were downloaded from the DepMap database (DepMap Public 23Q4 Primary Files). Gene expression and clinical data for TCGA-BRCA samples were retrieved using the TCGAbiolinks package, while BRCA sample data from the METABRIC cohort were obtained from cBioPortal. Breast cancer cases were classified as TNBC or non-TNBC based on the status of ER, PR, and HER2 receptors. The Lehmann subtype classification for TNBC was determined using a network-based tool (http://cbc.mc.vanderbilt.edu/tnbc/), which assigns signature scores for six subtypes (BL1, BL2, M, MSL, and LAR) to each TNBC patient.

### GO analysis of TNBC-specific SEs targets genes

Enrichment analysis was conducted on the set of TNBC-specific SE target genes to identify significantly overrepresented Gene Ontology (GO) biological processes (2023 release) using the bulk.geneset_enrichment function from Omicverse (https://github.com/Starlitnightly/omicverse). Following established research methods [15], these biological processes were categorized into the following groups: (i) DNA Repair: Includes processes such as double-strand break repair, DNA repair, and nucleotide-excision repair; (ii) Cell Cycle Transition: Encompasses processes like Cell Cycle, G1/S Transition, and G0 To G1 Transition; (iii) Protein Modification: Covers terms related to Protein Phosphorylation, Phosphatidylinositol, and Kinase activities; (iv) Signal Transduction: Involves signaling-related processes, including Signaling Molecules, Signaling Pathway, Cellular Process, Process, and Signal Transduction; (v) Transcriptional Regulation: Includes processes such as DNA-templated transcription, DNA-binding, Nucleic Acid-Templated activities, RNA Polymerase II function, and general Transcription regulation; (vi) Mesenchymal Development: Comprises processes related to tissue organ development, Differentiation, Migration, Adhesion, Mesenchymal development, and Stemness.

### NMF dimensionality reduction subtype

To apply non-negative matrix factorization (NMF) for dimensionality reduction on the H3K27ac signal matrix of TNBC-specific SE regions in TNBC cell lines, the expression matrix of TNBC-specific SE target genes in TNBC patients, and TNBC single-cell data, the ButchR R package was utilized. The analysis explored dimensionality from 2 to 6. Each matrix was decomposed into an exposure matrix (H-matrix) and a signature matrix (W-matrix). The optimal number of clusters was determined by evaluating the Cophenetic correlation coefficient, mean silhouette width, Frobenius error, and mean Amari distance. The Cophenetic correlation coefficient measures the consistency between the original distance matrix and the distance matrix derived from the hierarchical clustering of the NMF components, with higher values indicating better clustering stability. The mean silhouette width quantifies how similar each point is to its own cluster compared to other clusters, with values closer to 1 suggesting well-separated clusters. The Frobenius error assesses the reconstruction accuracy of the factorization, with lower values indicating better approximation of the original data. Finally, the mean Amari distance evaluates the stability of the cluster assignments by comparing the clusterings of different runs, with lower values signifying more consistent cluster results.

### Assessment of EMT score and status

A list of epithelial and mesenchymal markers was manually compiled from the literature [38]. The epithelial marker genes include CDH1, DSP, OCLN, and CRB3. The mesenchymal marker genes are VIM, CDH2, FOXC2, SNAI1, SNAI2, TWIST1, FN1, ITGB6, MMP2, MMP3, MMP9, SOX10, GSC, ZEB1, ZEB2, and TWIST2. Hybrid epithelial-mesenchymal transition (EMT) marker genes include PDPN, ITGA5, ITGA6, TGFBI, LAMC2, MMP10, LAMA3, CDH13, SERPINE1, P4HA2, TNC, and MMP1. The EMT score for each TNBC sample was calculated by subtracting the average RNA-seq z-score of mesenchymal marker genes from the average RNA-seq z-score of epithelial marker genes, following the method established by Chae et al. [39]. EMT status was then determined based on the expression profiles of epithelial, mesenchymal, and hybrid EMT marker genes. Samples were classified as epithelial if they predominantly expressed epithelial markers, as mesenchymal if they predominantly expressed mesenchymal markers, and as hybrid EMT if they expressed both epithelial and mesenchymal markers along with hybrid EMT markers.

### Gene sets enrichment analysis

To validate the characteristic biological processes associated with different NMF subtypes, we performed differential expression analysis and Gene Set Enrichment Analysis (GSEA) on the TCGA-TNBC and METABRIC-TNBC patient datasets. First, we retrieved the gene expression count matrix for TCGA-TNBC using TCGAbiolinks. Differential expression analysis was conducted using the dds.deg_analysis function in Omicverse with the DESeq2 method. For METABRIC-TNBC, differential analysis was performed using the Wilcoxon test. Next, we downloaded the Reactome_2022 gene set file from the Enrichr database and performed GSEA on the differentially expressed genes using the bulk.pyGSEA function in Omicverse, followed by result visualization.

### Tumor immune microenvironment assessment

The CIBERSORT and EPIC algorithms, available through the immunedeconv R package, were highly effective for analyzing the tumor immune microenvironment features across different NMF subtypes in TCGA-TNBC and METABRIC-TNBC patients. These algorithms estimated the composition and proportions of infiltrating immune cells and CAFs within the TME based on gene mRNA expression levels.

### TNBC scRNA-Seq analysis

TNBC scRNA-Seq datasets were obtained from the GEO database (GSE161529, GSE143423, GSE176078, GSE158673, GSE199515, and GSE180286). The datasets were processed and analyzed using Omicverse and Scanpy. The annotation of different scRNA-Seq datasets followed a structured workflow: (i) Quality Control: Data were filtered based on specific criteria: nUMIs < 500, genes < 250, and mitochondrial gene proportion > 5%; (ii) Data Normalization: The data were normalized, and the top 2000 highly variable genes were selected for downstream analysis; (iii) Dimensionality Reduction and Clustering: Principal component analysis (PCA) was performed, followed by clustering analysis; (iv) Cell Type Annotation: Cell groups were annotated using marker genes for various cell types, including B cells, T cells, endothelial cells, monocytes, stromal cells, and epithelial cells, based on established literature (Supplementary Table 2). Following annotation, tumor cells were isolated from the scRNA-seq data through the following steps: (i) Epithelial Cell Extraction: Epithelial cells were extracted from the various scRNA-seq datasets and integrated using the concat function; (ii) Integrated Data Processing: The integrated data underwent rigorous quality control, normalization, selection of highly variable genes, and PCA clustering, with batch effects mitigated using the Harmony algorithm; (iii) CNV Analysis: CNV analysis was conducted on the epithelial cells using infercnvpy, with normal breast tissue epithelial cells serving as a reference to derive CNV scores; (iv) Tumor Cell Identification: Based on CNV scores, cell groups were categorized into clusters by CNV status. Clusters with high CNV scores were identified as tumor cells, while those with low CNV scores were classified as normal epithelial cells. This structured approach ensures a robust and accurate annotation and isolation of tumor cells from scRNA-Seq datasets, facilitating further analysis of TNBC biology.

### SCSA malignant cell type annotation

To automate the annotation and classification of tumor cells, we used the pySCSA tool with specific parameter settings: celltype = cancer and target = cancersea. All other parameters were kept at their default values. The pySCSA tool automatically classified the tumor cells into the following categories: Stemness, DNA Repair, Metastasis, Differentiation, Inflammation, EMT, Cell Cycle, and Invasion.

### Pathway analysis with aucell

To conduct pathway enrichment analysis on TNBC tumor cells, we used the Reactome gene set with the AUCell method. We then analyzed differential pathway enrichment across the different NMF subtypes using the tl.rank_genes_groups function in Scanpy, applying the Wilcoxon statistical test. The results were visualized to highlight the top three significantly enriched pathways within each NMF subtype.

### CAFs subtype annotation

Stromal cell annotation across various TNBC scRNA-Seq datasets was conducted based on marker gene expression profiles specific to different types of CAFs. The categorization of CAF subtypes included: mCAFs (MMP11, POSTN, COL1A1, CDH11), iCAFs (CFD, PLA2G2A, CD34), vCAFs (MCAM, RGS5), tCAFs (ENO1, MME, NDRG1), ifnCAFs (IDO1), apCAFs (CD74, HLA-DRA), rCAFs (CCL19, CCL21), and dCAFs (MKI67). Subsequently, the _tl.rank_genes_groups function in Scanpy, employing the Wilcoxon statistical method, was used to identify feature genes characteristic of each CAF subtype. Feature genes were filtered based on predefined criteria (adjusted *p*-value < 0.05, score > 5, logFC > 1) to construct refined gene sets representing distinct CAF types. Using these gene sets, AUCell was employed to compute enrichment scores for tumor cells within the CAF gene set across TNBC scRNA-Seq datasets. Additionally, in the TCGA and METABRIC datasets, ssGSEA was used to calculate enrichment scores for TNBC patient samples within the CAF gene set. Lastly, correlation analyses were performed to assess the relationship between the H-matrix signatures of different NMF subtypes and the enrichment scores derived from the CAF gene set.

### Cell-cell communication analysis

The Cell2Cell algorithm was applied for cell-cell interaction analysis using TNBC scRNA-seq datasets GSE148673, GSE161529, GSE176078, and GSE199515. This analysis utilized default settings with 1000 permutations to quantitatively characterize and compare inferred cell-cell communications. The algorithm evaluates interactions based on the average expression levels of ligands and receptors across distinct cell populations.

### Spatial transcriptomics data analysis

Initially, spatial transcriptomics data from GSE213688, specifically samples GSM6592050 and GSM6592052 representing two TNBC samples, were retrieved. Using Scanpy, we normalized this data and selected the top 2000 highly variable genes. Subsequently, we employed the Scanorama algorithm to mitigate batch effects and integrate the spatial transcriptomics data. To annotate cell types within each spatial bin of the transcriptomics data, we integrated TNBC scRNA-Seq datasets GSE161529, GSE148673, GSE176078, and GSE199515 using Scanorama. Cosine distances between the Visium dataset and scRNA-Seq datasets facilitated the successful transfer of cell type annotations from scRNA-Seq to Visium data by comparing expression profiles across datasets. We then computed probability scores for different cell types in each bin, assigning cell type identities based on the highest score. To visualize associations between different NMF subtypes of tumor cells and various types of CAFs, we utilized Squidpy’s _gr.co_occurrence function to calculate the co-occurrence probabilities of different cell types within defined spatial distances. Additionally, the _gr.nhood_enrichment function was employed to analyze neighborhood enrichment scores, assessing spatial proximity relationships between these cell types. These analyses provided comprehensive insights into the spatial distribution and functional interactions between NMF subtypes of tumor cells and diverse CAF types.

### Core TFs regulome and TF activity

We utilized a list of 1,672 human transcription factors provided by the FANTOM5 project and integrated it with transcriptome data from METABRIC-TNBC patients. Using the Algorithm for the Reconstruction of Accurate Cellular Networks (ARACNe) [40, 41], we constructed a regulatory network of human transcription factors and their target genes. Through Virtual Inference of Protein Activity by Enriched Regulon (VIPER) analysis, we inferred the protein activity of transcription factors in different NMF subtypes (NES > 0 & *p*-value < 0.05), thereby identifying subtype-specific transcription factors and their gene regulatory networks. To identify SE-TF CRCs, we utilized the CRCmapper tool to analyze H3K27ac ChIP-seq data from TNBC cell lines [42]. In these CRCs, TFs regulated by SEs feedback to bind SE regions, forming a positive loop. By comparing the mesenchymal development subtype cell lines with the non-mesenchymal development subtype cell lines, we identified SE-TF CRCs uniquely present in the mesenchymal development subtype cell lines. To infer the core TFs of mesenchymal development subtype cell clusters in TNBC scRNA-Seq tumor cells, we first utilized the GRNBoost and RcisTarget functionalities in the pySCENIC tool [43]. Using the normalized expression matrix of tumor cells as input, we constructed a gene regulatory network of human TFs and inferred the activity of transcription factor nodes in the regulatory network. Subsequently, we employed the AUCell functionality to determine positively regulated TFs based on the inferred TF activity. Finally, we calculated the average activity of each TF in each NMF subtype, filtering out TFs with the highest average activity and an activity index greater than 0.2 in the mesenchymal development subtype. These TFs were identified as the core TFs of the mesenchymal development subtype.

### Knockdown sensitivity analysis of TFs

To further refine the core TFs, we utilized CRISPR gene knockout CERES scores for TNBC cell lines obtained from the DepMap database (DepMap Public 21Q3). In this scoring system, a score of 0 indicates that gene knockout has no significant impact on cell survival, while a score of -1 suggests a substantial effect on the survival of nearly all cell lines. Lower scores indicate a greater impact of the gene knockout on cell survival. We filtered TFs in the mesenchymal development subtype TNBC cell lines by selecting those with higher average expression levels compared to the non-mesenchymal development subtype TNBC cell lines and with lower average CERES scores than those in the non-mesenchymal development subtype cell lines.

### TNBC scRNA-Seq trajectories analysis

To investigate the expression patterns of TFs during pseudotime changes across different NMF subtypes, we employed the SEACells tool to perform Meta-cell calculation. This method reduces noise in the original dataset and enhances the accuracy of regression models. The approach identified 200 Meta-cells. Following this, we utilized the pyVIA tool with default parameters to infer pseudotime changes within the Meta-cells and analyze the expression dynamics of core TFs during this process.

### Cell culture

Human HEK293T, CAL51, MDA468, MDA231, MDA436, SUM159PT, HCC38, and BT549 cells, as well as mouse mammary carcinoma E0771 and 4T1 cells, were purchased from the American Type Culture Collection (ATCC, Manassas, VA). These cells were cultured in Dulbecco’s Modified Eagle’s Medium (DMEM) supplemented with 10% fetal bovine serum (FBS, Gibco) at 37 °C in a 5% CO₂ atmosphere. HCC1806 cells were maintained in RPMI-1640 medium with 10% FBS under the same conditions. All cell lines from ATCC were authenticated using short tandem repeat (STR) profiling, passaged for fewer than 10 passages, and routinely tested for Mycoplasma contamination.

### Plasmids, primers, transfection and viral particles infection

To delete a SE peak using CRISPR/Cas9, we designed a pair of guide RNAs (gRNAs) flanking the SE peak using the CRISPOR web tool. The gRNA targeting the SE proximal site was duplexed and cloned into the lentiCRISPR v2-puro vector (Addgene Plasmid #98290), while the gRNA targeting the SE distal site was cloned into the lentiCRISPR v2-blast vector (Addgene Plasmid #83480). These plasmids were then transfected into MDA468 and CAL51 cell lines. The excision of the SE fragment was verified by DNA blotting using two sets of primers: (i) An outer primer flanking the SE excision site; and (ii) An inner primer within the SE excision site. Successful excision of the SE should yield a fragment of approximately 1000 bp. Additionally, Western blotting was performed to confirm the downregulation of the target gene expression in cell lines with the excised SE fragment. To further investigate the functional role of VAX2 in TNBC, we employed RNA interference for gene knockdown and lentiviral transduction for gene overexpression. Short hairpin RNAs (shRNAs) for gene knockdown were inserted into the pLKO-Tet-On vector (Addgene Plasmid #21915). Human full-length VAX2 cDNA was subcloned into the psin-EF1-puro vector (Addgene Plasmid #61062) to generate stably transfected cells. Packaging plasmids PMD2.G (Addgene Plasmid #12259) and PsPAX2 (Addgene Plasmid #12260) were co-transfected with the LentiCRISPR v2-SE sgRNA, pLKO-Tet-On-VAX2 shRNA, or psin-EF1-puro-VAX2 overexpression plasmid into HEK293T cells. Viral particles were harvested 48 h post-transfection and used to infect target tumor cells in the presence of polybrene (8 µg/mL) for 24 h. Stably transfected cells were selected with puromycin or blasticidin. Primer sequences used for plasmid construction are listed in Supplementary Table 3.

### DNA extraction and Western blotting

DNA extraction was carried out using the Tiangen Biotech DNA Extraction Kit (#DP304) for both MDA468 and CAL51 cell lines. This procedure was also applied to MDA468 and CAL51 cells successfully infected with VAX2-SE269-proximal and distal sgRNA lentiviruses, following the kit’s instructions. Extracted DNA samples were amplified by PCR, supplemented with 10× DNA loading buffer, and diluted to a 1× concentration. Gel electrophoresis was performed in 1× TAE buffer. For immunoblotting, cells were harvested and lysed using either 1× SDS sample buffer or RIPA buffer (Cat#9806s, Cell Signaling Technology), with 1 mM phenyl- methanesulfonyl fluoride added immediately before use. Total protein (25–50 µg) was separated by SDS-PAGE, transferred to a PVDF membrane, and probed with specific antibodies as indicated.

### ChIP-qPCR and ChIP-Seq

Following the instructions of the SimpleChIP^®^ Enzymatic Chromatin IP Kit, DNA enriched by H3K27ac (GeneTex, # GTX128944) and BRD4 (CST, #13440) antibodies, along with input DNA, were extracted from MDA468, CAL51, and MDA231 cells. Additionally, DNA samples enriched by H3K27ac and BRD4 antibodies, as well as input DNA, were extracted from these cell lines after treatment with the drug JQ1 (TargetMol, #1268524-70-4) for 48 h. Quantitative PCR (qPCR) was performed using ChamQ SYBR qPCR Green Master Mix (Vazyme Biotech Co., Ltd., Nanjing, China Q311-03) and run on a Light Cycler 480 instrument (Roche Diagnostics). Primer sequences are listed in Supplementary Table 3. ChIP-Seq was performed using 10 × 10⁶ cross-linked MDA468 wild-type cells and VAX2-knockdown MDA468 cells. Sequencing libraries were prepared as described above. The following antibody was used for ChIP: Mouse anti-VAX2 (Santa Cruz, #sc-81422). ChIP-Seq libraries were sequenced on an Illumina HiSeq platform.

### Quantitative Real-Time PCR

Total RNA was isolated using the HiPure Universal RNA Mini Kit (Magen, R4130-03), and cDNA synthesis was carried out with the PrimeScript™ RT Reagent Kit with gDNA Eraser (Takara, RR047D). qRT-PCR was performed using ChamQ SYBR qPCR Green Master Mix (Vazyme Biotech, Q311-03) on a LightCycler 480 system (Roche Diagnostics). GAPDH was used as an internal reference to normalize target gene expression. All primers for these genes were listed in Supplementary Table 3.

### Cell clonogenic growth assay

Tumor cells were plated in 6-well plates. Plates were maintained in a 37 °C cell culture incubator with medium changes every 5 days. When distinct colonies became visible, cells were fixed with 4% formaldehyde at room temperature for 15 min, stained with 0.1% crystal violet for 40 min, washed with PBS, and photographed for quantification of colony formation using ImageJ software.

### Cell invasion and migration assay

Assays were performed in 24-well Boyden chambers (FALCON, 353097). Transwell insertscoated with growth factor reduced matrigel (BD Biosciences) for invasion assays. Transwell inserts had no Matrigel, designated for migration experiments. The prepared tumor cells (200 µL per well) were added to the upper chambers of the 24-well Boyden chambers inserts, with each cell strain plated in triplicate wells. The lower chambers were filled with 670 µL of DMEM medium containing 10% FBS. Plates were then placed in a cell culture incubator and incubated for 24 h. After incubation, the culture medium was removed, cells were fixed with 4% formaldehyde for 15 min, stained with 0.1% crystal violet for 40 min, and observed and photographed using a high-power inverted microscope. Quantitative analysis was performed using ImageJ software.

### Immunohistochemical staining

For the analysis of human TNBC breast tumors, this study selected 120 paraffin-embedded human TNBC breast lesion samples. These samples underwent histopathological and clinical diagnosis at the Sun Yat-sen University Cancer Prevention and Treatment Center. The samples were chosen from patients with available follow-up data, and no history of neoadjuvant therapy. Clinical information for the samples is summarized in Supplementary Table 4. All samples used in this study were approved by the Medical Ethics Committee of Sun Yat-sen University Cancer Prevention and Treatment Center. Sections were immersed in EDTA citrate buffer (pH 6.0 or pH 8.0) and heated in a microwave for antigen retrieval. Slides were then incubated overnight at 4 °C with anti-VAX2 (Santa Cruz, #sc-81422). Normal mouse IgG was used as a negative control to ensure specificity. Slides were then treated with HRP polymer-conjugated secondary antibody for 30 min and developed with diaminobenzidine solution (ZSGB-Bio). Nuclei were counterstained with hematoxylin. Image acquisition was performed using KFBIO scanning instruments. To assess VAX2 protein levels in patients, we first utilized the classifier function of the HALO platform to intelligently identify and accurately delineate tumor and non-tumor areas in the tissue sections. Subsequently, the CytoNuclear module was used to analyze the staining intensity and distribution of VAX2 in tumor cell nuclei, obtaining detailed histochemical scores (H-scores) at the nuclear level. Finally, patients were categorized into high-expression and low-expression groups based on the average H-scores of VAX2 staining intensity.

### Immunofluorescence staining

Mouse tumor samples were immersed in cold 2-methylbutane (Sigma-Aldrich, #277258) for OCT embedding and stored at -80 °C. Tissue blocks were sectioned into 10 mm thick slices and mounted on glass slides. The sections were air-dried and fixed in cold 100% methanol. The slides were rehydrated in 1× PBS, permeabilized with 0.2% Triton X-100 (Thermo Fisher, #HFH10) in PBS at room temperature (RT) for 3 h, and blocked with 10% normal donkey serum (NDS) (Abcam, #ab7475) in PBS at RT for at least 1 h. After removing the blocking buffer, the tissues were incubated overnight at 4 °C with a mixture of primary anti-αSMA (Thermo Fisher, #MA5-11547) and anti-CDH11 (Thermo Fisher, #71-7600) antibodies. At RT, the slides were washed three timeson a shaker with 0.2% Tween-20 (Solarbio, #T8220) in PBS, then incubated with a mixture of secondary anti-mouse (Invitrogen, #A11029) and anti-rabbit (Invitrogen, #A11012) antibodies and DAPI (Sigma-Aldrich, D9542-1MG) for 1 h at RT. The slides were then washed again three times on a shaker with 0.2% Tween-20 in PBS at RT, and if applicable, incubated with a conjugated antibody mixture for 1 h at RT. In the final step, the slides were washed three times on a shaker with 0.2% Tween-20 in PBS at RT, mounted with Dako Fluorescence Mounting Medium (Sigma-Aldrich, #F4680), and covered with a coverslip. Image acquisition was performed using KFBIO scanning instruments with a 40× objective. HALO software was used to analyze the fluorescent regions of the tissues. For each sample, a region of interest was manually defined to exclude tissue edges and artifacts (e.g., signals caused by tissue folds, dust particles, or bubbles). The HIGH-PLEX FL analysis module automatically identified regions of different colored cell populations and calculated the area proportions.

### IC50 assay

Tumor cells were seeded into 96-well plates. After cell adherence, the culture medium was removed, and 200 µL of culture medium containing various concentrations of BET inhibitors (TargetMol, I-BET-762, OTX015, and JQ1) was added. The concentration gradient included 10 µM, 5 µM, 2.5 µM, 1.25 µM, 0.625 µM, 0.3125 µM, and 0 µM. The 96-well plates were incubated at 37 °C for 48 h. After the 48-hour incubation period, 15 µL of MTT solution at a concentration of 5 mg/mL was added to each well and incubated for an additional 4 h. Following the MTT incubation, the culture medium was removed, and 150 µL of DMSO was added to each well. Absorbance was measured at 470 nm using a microplate reader. Changes in absorbance were used to reflect the survival of tumor cells under different drug concentrations. Finally, IC50 curves were plotted using GraphPad.

### Animal treatment protocol

C57BL/6 mice, BALB/c mice, and BALB/c nude mice were purchased from the Guangdong Animal Experimental Center and were 6 weeks old. All procedures involving mice and experimental protocols were approved by Institutional Animal Care and Use Committee (IACUC) of Sun Yat-sen University Cancer Center. E0771-Control and E0771-shVAX2 tumor cells were inoculated into C57BL/6 mice, while 4T1-Control and 4T1-shVAX2 tumor cells were inoculated into BALB/c mice. In BALB/c nude mice, MDA231-Control and MDA231-VAX2 overexpression cells were injected. Similarly, CAL51-Control, CAL51-sgSE, and CAL51-shVAX2 tumor cells were injected into BALB/c nude mice. Following inoculation, tumor size and volume in C57BL/6 and BALB/c mice were measured weekly from the first week until the end of the experiment, while for BALB/c nude mice bearing MDA231 tumors, monitoring began immediately after inoculation. In the CAL51 model, drug administration commenced when tumors reached approximately 100 mm³, at which point the BALB/c nude mice were randomly divided into six groups: CAL51-Control + JQ1, CAL51-Control + DMSO, CAL51-sgSE + JQ1, CAL51-sgSE + DMSO, CAL51-shVAX2 + JQ1, and CAL51-shVAX2 + DMSO. JQ1 was administered intraperitoneally at 50 mg/kg per mouse (100 µL per dose) for 10 consecutive days, with strict control over injection timing and dosage to ensure consistency. JQ1 was dissolved in a vehicle solution containing 10% DMSO, 10% PEG300, 20% Tween80, and 60% ddH₂O.

### RNA-Seq analysis

In a six-well plate, MDA468-Control cells and MDA468-shVAX2 cells were cultured in three replicate wells each. Upon reaching 80% confluence, the culture medium was removed, and the cells were washed with 1× PBS. TRIzol reagent (Thermo Fisher, #15596018) was then added to each well (1 mL per well), and the cells were lysed by thorough pipetting. The TRIzol solution was transferred to 1.5 mL Eppendorf tubes and stored at -80 °C for subsequent sequencing. RNA-Seq was performed by Novogene, producing fastq files. Quality control of the data was conducted using FastQC and Trim-Galore. After quality control, the data were aligned to the human Hg19 genome using HISAT2. Gene expression counts were then extracted using featureCounts. Differential expression analysis was carried out using the dds.deg_analysis function in Omicverse, applying the DESeq2 method. Subsequently, GSEA was performed using the bulk.pyGSEA function in Omicverse, focusing on Reactome_2022 pathways and TNBC mesenchymal development subtype signature genesets.

### Machine learning models

To validate the predictive value of VAX2.Sig, various machine learning classification models from Scikit-Learn were employed (Supplementary Table 5). The models selected for evaluation included logistic regression, stochastic gradient descent (SGD) classifier, support vector classification (SVC), k-nearest neighbors classification (KNN), radius-based neighbors classification (RadiusNN), decision tree classifier (DecisionTree), gradient boosting classifier (GradientBoost), histogram-based boosting classifier (HistGradientBoost), random forest classifier (RandomForest), nu-support vector classification (NuSVC), extremely randomized trees classifier (ExtraTrees), multi-layer perceptron classifier (MLP), and Gaussian process classification (GaussianProcess). Each model underwent 3-fold cross-validation on the training cohort and was then evaluated on the test cohort. Model performance was assessed based on the Area Under the Curve (AUC) for both the training and test cohorts, with the optimal model selected based on these metrics. An additional validation cohort was used to further confirm the predictions of the best-performing model.

### Statistical analysis

All statistical analyses were performed using R (version 4.2.2). The Wilcoxon rank-sum test was used for comparisons between two groups of continuous variables, while the Kruskal-Wallis test was applied for comparisons involving three or more groups. Survival curves were generated using the Kaplan-Meier method, and differences were assessed with the log-rank test. A *p*-value < 0.05 was considered statistically significant.

## Results

### The TNBC super-enhancer landscape differs significantly from non-TNBC and normal mammary epithelium

**Fig. 1:**
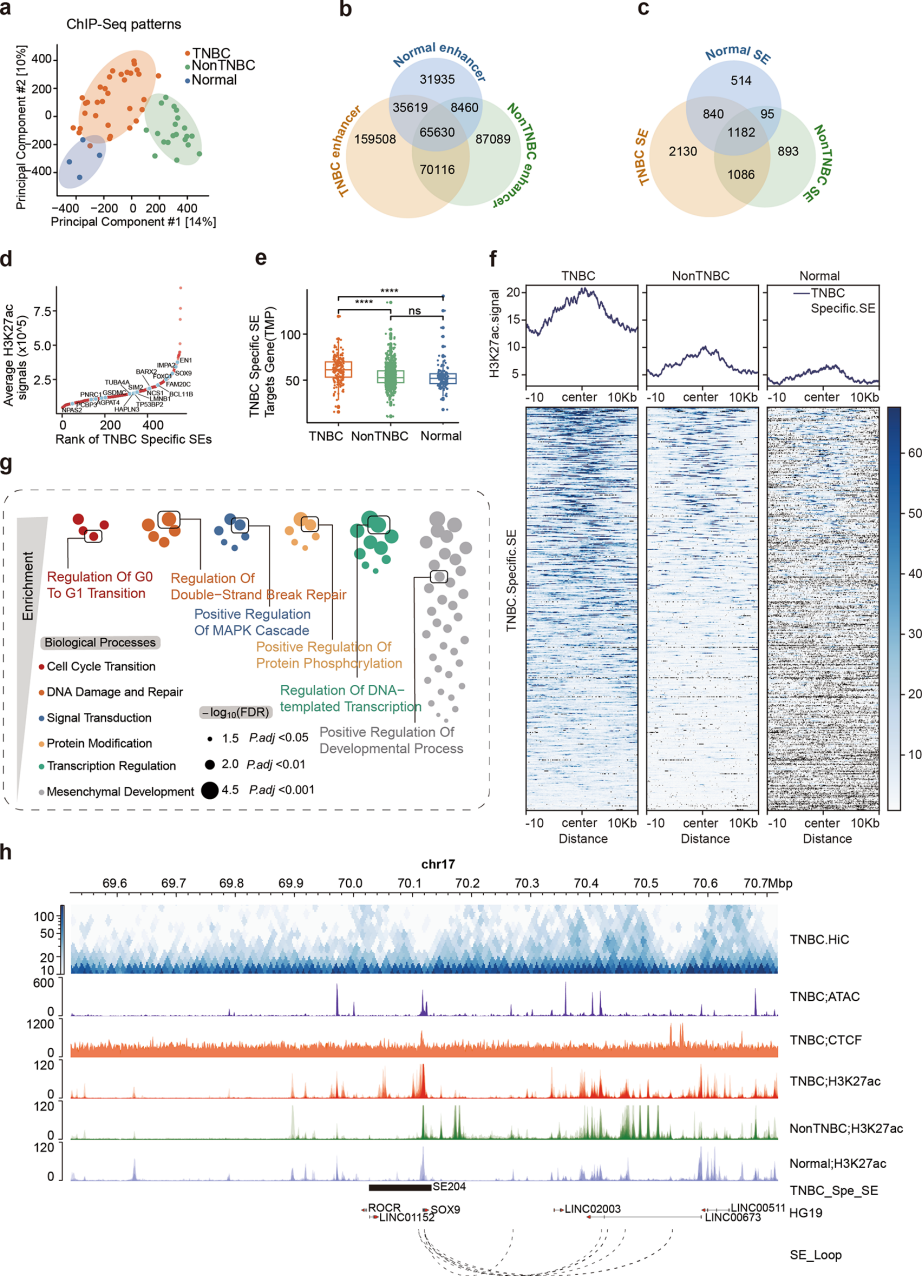
Triple-negative breast cancer-specific super-enhancer profile. (**a**) Multidimensional scaling plot for global H3K27ac ChIP-seq patterns in breast cancer. (**b**) Venn diagram showing overlaps in the total number of H3K27ac-defined active enhancer peaks in TNBC (yellow), non-TNBC (green) and normal mammary epithelium (blue). (**c**) Same as (**b**), analyzing the number of active SE peaks. (**d**) Distribution of TNBC-specific SE signals and corresponding target genes. (**e**) Boxplot representing the mean expression of TNBC-specific SE target genes among TNBC, non-TNBC, and normal mammary epithelium. (**f**) Genome-wide rank-ordered heatmap of mean H3K27ac ChIP-seq signal at TNBC-specific SE peaks. (**g**) Enrichment analysis of TNBC-specific SE target genes. Individual terms are grouped into meta-functional classes. FDR, false discovery rate. (**h**) Multiple layers of regulatory information, including HiC, CTCF, ATAC–seq and H3K27ac ChIP–seq profiles, TNBC-specific SEs and chromatin interactions, integrated in this study, are shown exemplarily for the SOX9 locus, which is regulated by one of the TNBC-specific SEs. Statistical analysis was performed using the Wilcoxon rank-sum test. **** *p* < 0.0001.

To comprehensively elucidate the landscape of enhancers and SEs in TNBC and their biological contributions, we analyzed the quality-controlled H3K27ac ChIP-seq datasets derived from TNBC samples (*n* = 10), TNBC cell lines (*n* = 15), non-TNBC cell lines (*n* = 11) and normal mammary epithelium cell lines (*n* = 3) (Supplementary Fig. 1a-b and Supplementary Table 1). PCA of H3K27ac signals revealed distinct patterns in TNBC samples and cell lines, forming unique clusters separate from non-TNBC and normal mammary epithelium (Fig. 1a). This pattern highlights the distinct TNBC enhancer and SE profiles.

To determine the distinct enhancer and SE landscape in TNBC, we performed an overlap analysis to summarize the shared enhancers and SEs across all TNBC samples and cell lines, comparing them with those shared in non-TNBC and normal mammary epithelium (Supplementary Fig. 1c). Our analysis revealed that TNBC harbors a greater number of enhancers and SEs than non-TNBC and normal mammary epithelium. Specifically, the number of TNBC-specific enhancers (*n* = 159,508) far exceeded that of non-TNBC-specific enhancers (*n* = 87,089) and normal-specific enhancers (*n* = 31,935), suggesting extensive enhancer acquisition in TNBC (Fig. 1b). A similar trend was observed for SEs, with TNBC-specific SEs (*n* = 2,130) markedly outnumbering those in non-TNBC (*n* = 893) and normal tissue (*n* = 514) (Fig. 1c). These findings underscore the marked divergence in enhancer and SE profiles among TNBC, non-TNBC, and normal mammary epithelium, highlighting the critical role of acquired enhancers and SEs in TNBC malignancy.

Compared to typical enhancers, SEs are crucial for maintaining cancer cell identity and driving oncogenic transcription. We concentrated on the significance of TNBC-specific SEs in promoting TNBC malignancy. Specifically, we identified 555 TNBC-specific SEs that were consistently present in at least three TNBC samples or cell lines, reducing potential biases from individual samples or cell lines (Supplementary Fig. 1c and Supplementary Table 6). These TNBC-specific SEs were ranked by their median H3K27ac signal across all samples and cell lines, and the corresponding target genes were annotated using a prioritization approach integrating Hi-C interaction data, H3K27ac signal intensity, and genomic localization. We compiled a comprehensive list of genes associated with TNBC-specific SE targets. Among these genes are EN1, SOX9, FOXC1, BARX2, LMNB1, and BCL11B (Fig. 1d and h), which have been highlighted in previous studies [44], affirming the robustness and reproducibility of our analysis. Analysis of two independent breast cancer RNA-seq datasets revealed significant upregulation of TNBC-specific SE target genes in TNBC compared to non-TNBC and normal breast tissues in the TCGA-BRCA and METABRIC datasets (Fig. 1e and Supplementary Fig. 1d). Furthermore, the heatmap and H3K27ac signal profiles demonstrated higher enrichment of active signals in TNBC-specific SEs, compared to non-TNBC and normal mammary epithelium cell lines (Fig. 1f), further confirming the accuracy and specificity of our identification of TNBC-specific SEs.

To further understand the biological functions of TNBC-specific SEs, we conducted an enrichment analysis of their target genes, identifying significantly enriched GO biological processes. Consistent with previous research methodologies [15], these biological processes were categorized into distinct functional groups, primarily linked to (i) cell cycle transition, (ii) DNA repair, (iii) signal transduction, (iv) protein modification, (v) transcriptional regulation, and (vi) mesenchymal development (Fig. 1g). In summary, our analysis revealed the unique enhancer and SE landscapes in TNBC, identifying key TNBC-specific SEs and their target genes, which likely drive the malignant processes characteristic of TNBC biology.

### Heterogeneity subtyping of TNBC-specific SE and target genes

**Fig. 2:**
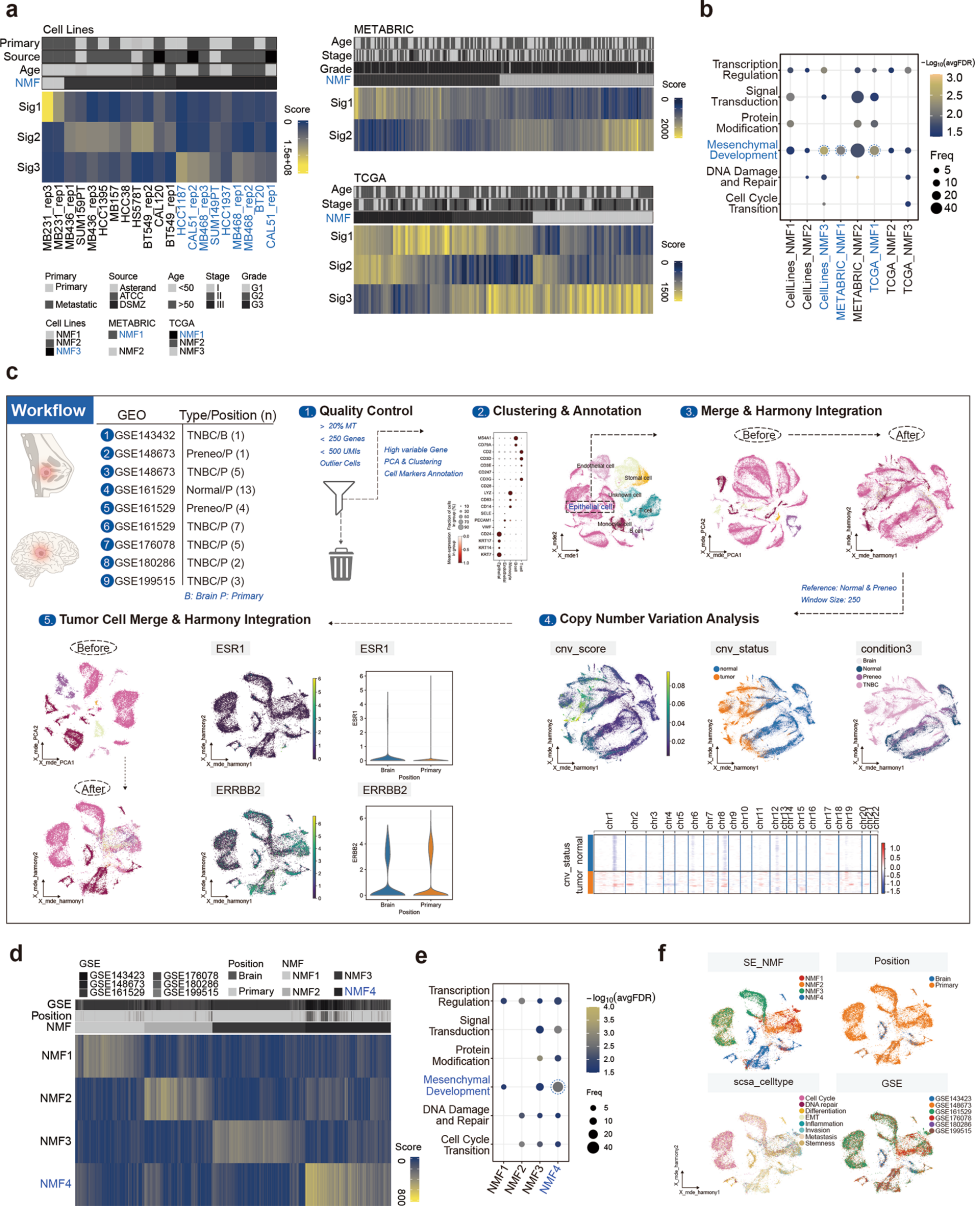
Heterogeneous subtype defined by TNBC-Specific SEs. (**a**) NMF analysis of TNBC-specific SE H3K27ac signals in TNBC cell lines and NMF analysis of TNBC samples based on the expression of the TNBC-specific SE target genes. (**b**) Enrichment analysis of signature genes from different NMF subtypes based on TNBC-specific SE H3K27ac signals or target genes. The enrichment analysis results were categorized into different functional groups. (**c**) Workflow for extracting TNBC tumor cells from scRNA-Seq datasets. (**d**) NMF analysis of TNBC tumor cells based on the expression of the TNBC-specific SE target genes. (**e**) Enrichment analysis of signature genes from different NMF subtypes based on TNBC-specific SE target genes in TNBC tumor cells. (**f**) Dataset sources, lesion locations, SCSA functional subtypes, and different NMF subtypes of TNBC tumor cells.

To investigate the impact of TNBC-specific SEs on TNBC heterogeneity, we applied NMF as a dimensionality reduction method for the H3K27ac signal matrix from TNBC-specific SE regions in TNBC cell lines and to the expression matrix of the TNBC-specific SE target genes in TNBC samples from the TCGA and METABRIC datasets (Supplementary Table 7). Utilizing NMF on 15 TNBC cell lines, 121 TNBC patients in the TCGA-BRCA dataset, and 206 TNBC patients in the METABRIC dataset, we determined that the optimal number of subtypes was 3, 3, and 2, respectively, based on NMF clustering evaluation metrics (Fig. 2a and Supplementary Fig. 2a). In TNBC cell lines and patient samples, distinct subtypes exhibited different SE signals and SE target gene signature activities (Fig. 2a). These SE target gene signatures across different subtypes were involved in significantly different biological processes, indicating substantial heterogeneity of TNBC-specific SEs within TNBC (Fig. 2b and Supplementary Fig. 2b-d). In both TNBC cell lines and patient samples, one consistent subtype was characterized by biological functions related to mesenchymal development, highlighting the distinctive role of TNBC-specific SEs in promoting the mesenchymal development subtype within TNBC.

Bulk RNA-Seq faces challenges in differentiating between tumor and stromal cells, affecting classification precision. Recent TNBC scRNA-Seq studies revealed significant tumor cell heterogeneity, indicating that bulk RNA-Seq may miss this complexity. Integrating scRNA-Seq data should be performed to better understand the impact of TNBC-specific SE target gene expression on TNBC heterogeneity. In this study, we collected scRNA-Seq datasets from TNBC patient samples, including primary tumors and brain metastases. By performing rigorous quality control, dimensionality reduction, clustering, and cell annotation, we isolated epithelial cells and identified tumor cells using copy number variation analysis. Finally, we integrated these data to create a comprehensive dataset of 28,317 TNBC tumor cells (Fig. 2c and Supplementary Table 8). After performing NMF dimensionality reduction and clustering based on TNBC-specific SE target gene expression, these cells were classified into four distinct subtypes, each exhibiting significant differences in characteristics and biological functions (Fig. 2d and Supplementary Fig. 2e). Similar to the subtypes identified in TNBC cell lines and bulk RNA-based NMF subtypes, we identified a subtype characterized by mesenchymal development functions (Fig. 2e-f and Supplementary Fig. 2f).

### TNBC-specific SE heterogeneity analysis reveals a consistent high-risk mesenchymal development subtype

**Fig. 3:**
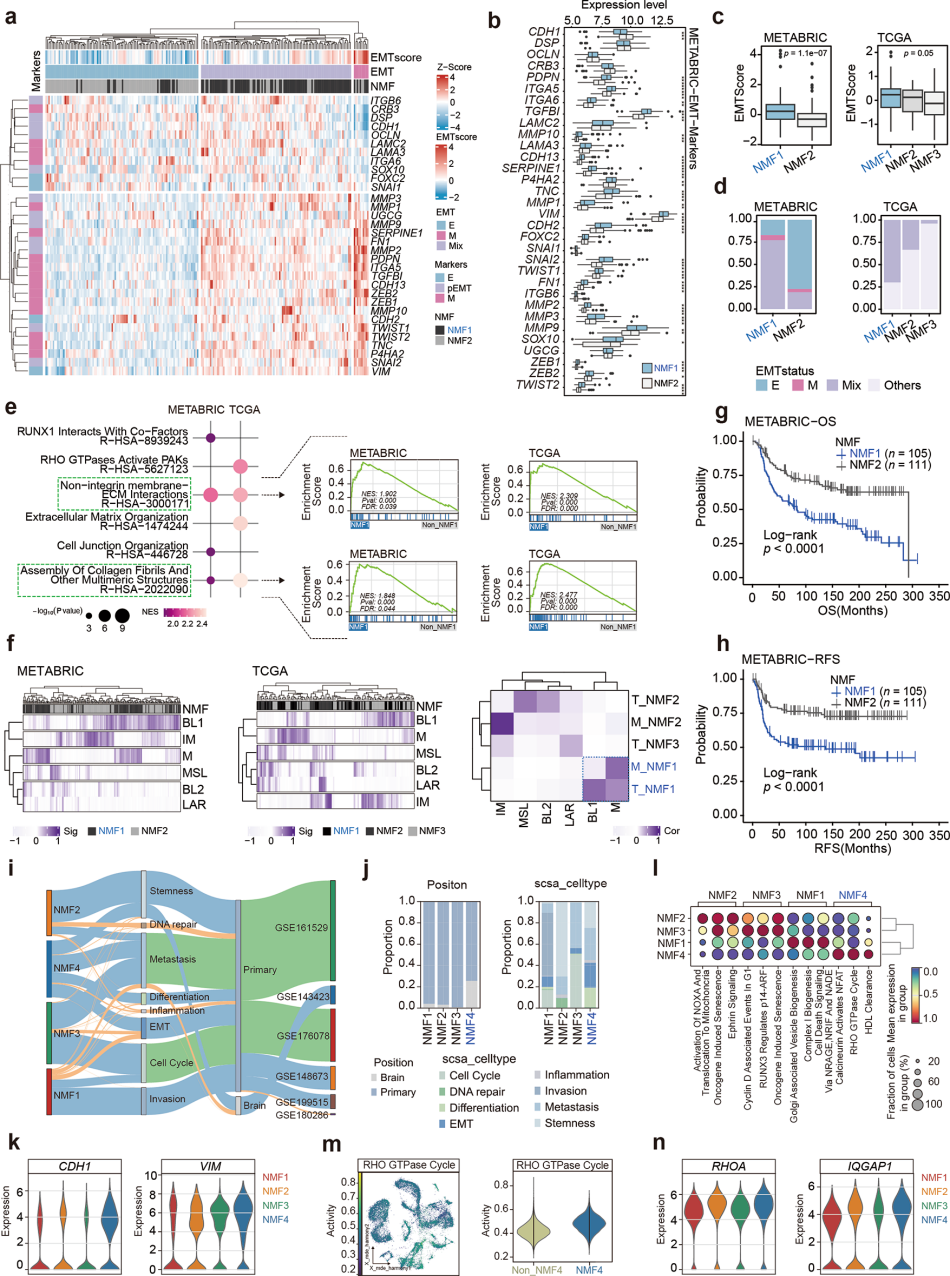
TNBC-specific SE heterogeneity analysis identifies a consistently high-risk mesenchymal development subtype. (**a–d**) EMT scores and status across different TNBC NMF subtypes (**a**). Mean expression levels of EMT marker genes (**b**), EMT scores (**c**), and the distribution of different EMT statuses (**d**) across TNBC NMF subtypes. (**e**) GSEA of differentially expressed genes between mesenchymal and non-mesenchymal TNBC subtypes in METABRIC and TCGA cohorts. (**f**) Lehmann bulk RNA-seq subtype gene signatures in TNBC samples from METABRIC (left) and TCGA (mid). The correlation between Lehmann bulk RNA-seq subtype gene signatures and NMF subtype gene signatures (right). (**g-h**) Kaplan–Meier OS (**g**) and RFS (**h**) curves for patients assigned to different NMF subtypes in METABRIC. (**i-j**) River plot and bar plot showing an overview of dataset sources, lesion locations, SCSA functional subtypes, and different NMF subtypes of TNBC tumor cells. (**k**) Expression of EMT marker genes CDH1 and VIM across different NMF subtypes of TNBC tumor cells. (**l**) Pathway enrichment analysis of different NMF subtypes of TNBC tumor cells using AUCell. (**m-n**) Enrichment of Rho GTPase cycle pathway activity (**m**) and the expression of corresponding marker genes (**n**) across different NMF subtypes of TNBC tumor cells. Statistical analysis was performed using the Wilcoxon rank-sum test. * *p* < 0.05, ** *p* < 0.01, *** *p* < 0.001, ****p < 0.0001.

The consistent identification of the mesenchymal development subtype across TNBC cell lines, clinical samples, and single-cell analyses emphasizes this subtype’s stability and uniqueness within SE heterogeneity classification. The consistent observation underscores the necessity of further investigation into the tumor biology associated with this subtype. EMT is an important process in the conversion of epithelial to mesenchymal state, significantly contributing to mesenchymal development and tumor progression. Our evaluation of TNBC clinical samples revealed that the mesenchymal development subtype showed a hybrid EMT state (Fig. 3a and d, Supplementary Fig. 3a), characterized by significant upregulation of epithelial, partial EMT, and mesenchymal marker genes (Fig. 3b and Supplementary Fig. 3b), as well as an elevated EMT score (Fig. 3c). To explore the underlying mechanisms, we conducted differential gene expression and GSEA, revealing significant enrichment of ECM-related pathways, such as integrin-ECM interactions, collagen fibers, and polymer structures, in the mesenchymal development subtype (Fig. 3e). The ECM, mainly produced by CAFs within the TME, plays a crucial role in the EMT process [45]. Immune infiltration analysis further revealed that this subtype exhibited an increased proportion of CAFs and a decreased proportion of immune cells (Supplementary Fig. 3c).

Additionally, we examined the relationship between the TNBC mesenchymal development subtype and bulk RNA-defined molecular subtypes, as well as their clinical implications. According to the classification of TNBC molecular subtypes by Lehmann et al. [6], the mesenchymal development subtype exhibited mixed characteristics, predominantly exhibiting features of the mesenchymal (M) subtype, followed by traits of the basal-like 1 (BL1) subtype (Fig. 3f and Supplementary Table 7). The M subtype is closely associated with cell motility, differentiation, growth factor signaling, and EMT, whereas the BL1 subtype is primarily linked to cell cycle and proliferation mechanisms [6]. Clinical prognostic analysis revealed that patients with the M subtype had poor survival outcomes and a low response rate to chemotherapy [6]. In the METABRIC NMF subtype classification, patients within the mesenchymal development subtype exhibited poor overall survival (OS) and recurrence-free survival (RFS) (Fig. 3g and h). Multivariate Cox regression analysis further confirmed that the mesenchymal development subtype is an independent high-risk factor (Supplementary Fig. 3d and e). In the TCGA-TNBC dataset, although survival differences among various NMF subtypes did not achieve statistical significance, the survival trend for patients in the mesenchymal development subtype was notably worse (Supplementary Fig. 3f). These findings suggest that the TNBC-specific SE-driven mesenchymal development subtype is associated with a higher risk of malignancy.

Similar malignant characteristics were observed in the TNBC single-cell mesenchymal development subtype. Firstly, analysis of tumor cell origin indicated a higher proportion of tumor cells from brain metastatic lesions in the mesenchymal development subtype (Fig. 3i and j). Additionally, utilizing the SCSA tool, we analyzed the characteristics of different NMF subtypes in TNBC tumor cells, including cell cycle, DNA repair, differentiation, EMT, inflammation, invasion, metastasis, and stem cell-related traits, and showed that cells exhibiting EMT characteristics predominantly found in the mesenchymal development subtype (Fig. 3i and j). Expression analysis of EMT-related marker genes revealed elevated levels of the epithelial marker gene CDH1 and the mesenchymal marker gene VIM within this subtype (Fig. 3k and Supplementary Fig. 3g). Furthermore, AUCell pathway enrichment analysis identified significant enrichment of the Rho GTPase cycle in the mesenchymal development subtype, accompanied by higher expression levels of effector molecules RHOA and IQGAP1 (Fig. 3l-n). The Rho GTPase cycle is known to promote ECM formation and EMT [46]. These results support the association of the mesenchymal development subtype of TNBC with increased malignancy.

### ECM-enriched tumor microenvironment characteristics of the mesenchymal development subtype TNBC

**Fig. 4:**
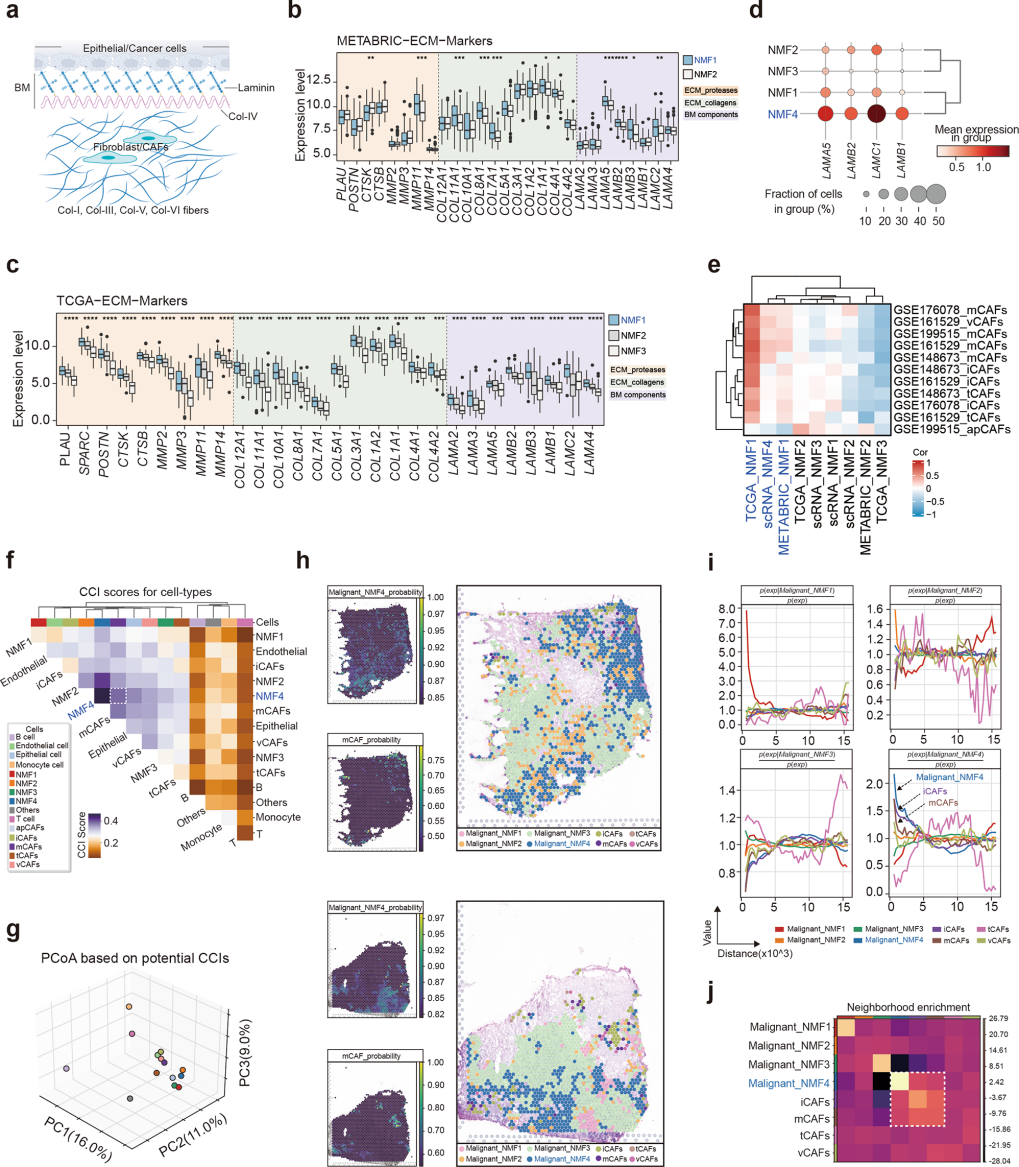
Tumor microenvironment characteristics of the mesenchymal development subtype. (**a**) Schematic representation of sources and components of the ECM. (**b–d**) Expression of ECM marker genes across different NMF subtypes in METABRIC (**b**), TCGA (**c**), and scRNA-seq (**d**). (**e**) Correlation between the signature gene enrichment scores of different CAF subtypes and the signature scores of NMF subtypes. (**f-g**) Cell-cell communication interaction scores between different cell types in the tumor microenvironment using the Cell2Cell tool. (**h**) Spatial images showing cell abundance and location, as estimated by Scanpy, for different NMF subtype tumor cells and different CAF subtypes. (**i**) Spatial distances between different NMF subtype tumor cells and different CAF subtypes. (**j**) Neighborhood enrichment analysis between different NMF subtype tumor cells and different CAF subtypes. Statistical analysis was performed using the Wilcoxon rank-sum test. * *p* < 0.05, ** *p* < 0.01, *** *p* < 0.001, ****p < 0.0001.

The ECM mainly consists of the basement membrane (BM) and interstitial matrix (Fig. 4a). The basement membrane is woven from laminin and type IV collagen, further cross-linking with nidogens and basement membrane proteoglycans, among other proteins. These components are primarily produced by epithelial cells, endothelial cells, and stromal cells. Basement membrane components on the basal side of epithelial cells are crucial for maintaining cell polarity from apical to basal. The interstitial matrix is primarily produced by fibroblasts, with a small amount also coming from endothelial or epithelial cells. Modifying extracellular matrix stiffness within the interstitial matrix can induce malignant phenotypes in tumor cells. In summary, the ECM, through the microenvironment it forms, influences the polarity, morphology, and cellular phenotype of tumor cells, thereby affecting their invasive and metastatic capabilities.

To further investigate the unique characteristics of the mesenchymal development subtype in TNBC, we first analyzed the gene expression profiles related to the BM and interstitial matrix components in different NMF subtypes of TNBC from the METABRIC and TCGA datasets. The results revealed elevated expression levels of BM-related genes (e.g., LAMA5, LAMB2, LAMB3, and LAMC2), extracellular matrix collagens (e.g., COL11A1, COL8A1, COL7A1, COL1A1, and COL4A1), and extracellular matrix enzymes (e.g., CTSK and MMP11) in the mesenchymal development subtype (Fig. 4b and c). scRNA-seq validation confirmed the elevated expression of BM components in this subtype (e.g., LAMA5, LAMB1, LAMB2, LAMC1) (Fig. 4d and Supplementary Fig. 4a). Additionally, immune infiltration analysis revealed the mesenchymal development subtype exhibited a higher proportion of infiltrating CAFs, which promote ECM formation (Supplementary Fig. 3c). Next, we explored the relationship between tumor cells and CAFs across different NMF subtypes. We curated feature genes representing various types of CAFs from diverse TNBC scRNA-seq datasets and compiled a comprehensive gene set (Supplementary Fig. 4b-c and Supplementary Table 9). By assessing the enrichment of various NMF subtypes within this CAF-related gene set and correlating their H-matrix signatures with the enrichment score of the CAF gene set, we found a positive association between the mesenchymal development subtype and CAFs, particularly mCAFs, which are primarily involved in ECM formation (Fig. 4e).

Next, we analyzed scRNA-seq and spatial transcriptomic RNA-seq (stRNA-seq) to more intuitively characterize the TME features of the mesenchymal development subtype. Initially, we used the cell2cell tool, which quantifies and compares inferred cell-cell communication networks based on the average expression levels of ligands and receptors in cell populations. We analyzed the GSE161529 TNBC scRNA-seq dataset due to its large cell count and rich representation of tumor cells and CAFs. Our investigation revealed significant interactions between tumor cells of different NMF subtypes and mCAFs, with the most prominent interaction occurring between the mesenchymal development subtype tumor cells and mCAFs, as indicated by their closer spatial Euclidean distances (Fig. 4f and g). Similar results were observed in three other TNBC scRNA-Seq datasets (GSE148673, GSE176078, and GSE199515) (Supplementary Fig. 4d-f). Moreover, stRNA-seq analysis illustrated the close association between the mesenchymal development subtype tumor cells and mCAFs in two TNBC samples (Fig. 4h). In regions of closer spatial proximity, the mesenchymal development subtype tumor cells and mCAFs exhibited a higher likelihood of co-occurrence (Fig. 4i). Neighboring enrichment analysis revealed that mCAFs had a higher neighbor enrichment score with the mesenchymal development subtype cells compared to other tumor cell types (Fig. 4j). Collectively, these results indicated the spatial proximity and intensive interaction between the mesenchymal development subtype tumor cells and mCAFs.

### Master regulator regulated by TNBC-specific SE in mesenchymal development subtype

**Fig. 5:**
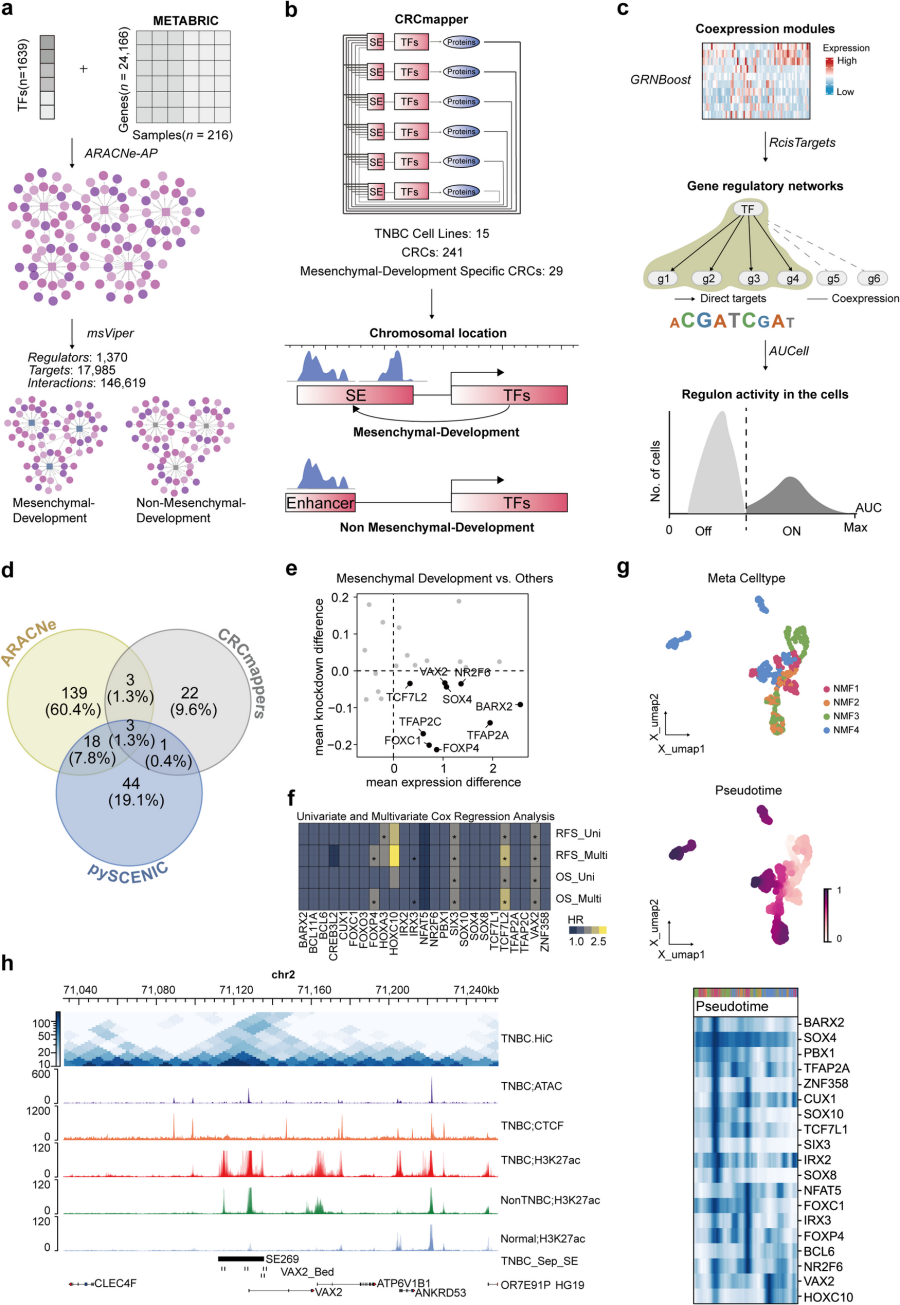
The master regulator for the mesenchymal development subtype of TNBC. (**a–c**) Workflow for determining the core TFs and computing TF activities using the ARACNe-AP and msVIPER methods (**a**), CRCmapper (**b**), and the pySCENIC algorithm (**c**). CRC, core regulatory circuit. (**d**) Venn diagram illustrating the core TFs identified by different algorithms. (**e**) Comparison of the knockdown sensitivity of all core TFs (n = 25 genes) between mesenchymal development subtype cell lines (n = 6) and other cancer cell lines (n = 9). The labeled genes are those that are highly specific for mesenchymal development subtype cell lines. (**f**) Univariate and multivariate Cox analyses of the effects of core TFs on TNBC survival. * *p* < 0.05. (**g**) Changes in the expression levels of core TFs during single-cell pseudotime analysis. (**h**) Integration of multiple layers of regulatory information, including Hi-C, CTCF, ATAC-seq, H3K27ac ChIP-seq profiles, TNBC-specific SEs, and chromatin interactions, exemplified by the VAX2 locus, a master regulator controlled by TNBC-specific SEs.

The dysregulation in transcription driven by SEs interacting with the master regulators is a key mechanism underlying tumor malignancy. To identify the key master regulator driving the mesenchymal development subtype, we applied the ARACNe [40], CRCmapper [42], and pySCENIC [43] algorithms on the METABRIC-TNBC, TNBC cell line, and TNBC scRNA-seq datasets, respectively (Fig. 5a-c). The ARACNe and pySCENIC algorithms identified core TFs with abnormal transcriptional regulatory activity in the mesenchymal developmental subtype. Simultaneously, the CRCmapper algorithm pinpointed the SEs and TFs that constitute CRCs within the subtype. Notably, the TFs within these CRCs are not only regulated by SEs but also participate in a feedback loop by binding to SEs, thereby forming a feedforward mechanism. In the METABRIC-TNBC dataset, we identified 163 core TFs; in TNBC cell lines, 29 CRCs were identified; and in TNBC scRNA-Seq data, we identified 68 core TFs (Supplementary Fig. 5a-c and Supplementary Table 10). Among these, 25 core TFs were consistently identified by at least two algorithms, with three TFs—VAX2, SOX10, and BARX2—being consistently recognized across all three algorithms (Fig. 5d).

To identify the master regulator among the 25 core TFs identified by at least two algorithms, we performed a multi-step analysis. First, we assessed the CERES scores and mRNA expression levels of these core TFs in TNBC cell lines. Nine TFs exhibited elevated mRNA expression in the mesenchymal development subtype and significantly impacted cell viability (Fig. 5e). Next, we evaluated the clinical significance of these TFs. Among them, only SIX3, TCF7L2, and VAX2 were identified as independent prognostic factors in the METABRIC-TNBC dataset (Fig. 5f and Supplementary Fig. 5d). Finally, pseudo-temporal analysis of TNBC scRNA-seq data revealed significant temporal variations in most TFs, except SOX4 (Fig. 5g). Our comprehensive analysis prioritized VAX2 and TCF7L2. Considering that TCF7L2 is not a target gene regulated directly by TNBC-specific SEs, while VAX2 is, and that VAX2 is specifically expressed in the mesenchymal development subtype of TNBC cell lines, we concluded that VAX2, regulated by TNBC-specific SE269 (chr2: 71,112,006–71,135,503), is the key master regulator driving the development of the mesenchymal development subtype of TNBC (Fig. 5h and Supplementary Fig. 5e).

### Experimental verification of the core role of the master regulator-VAX2 in themesenchymal development subtype

**Fig. 6:**
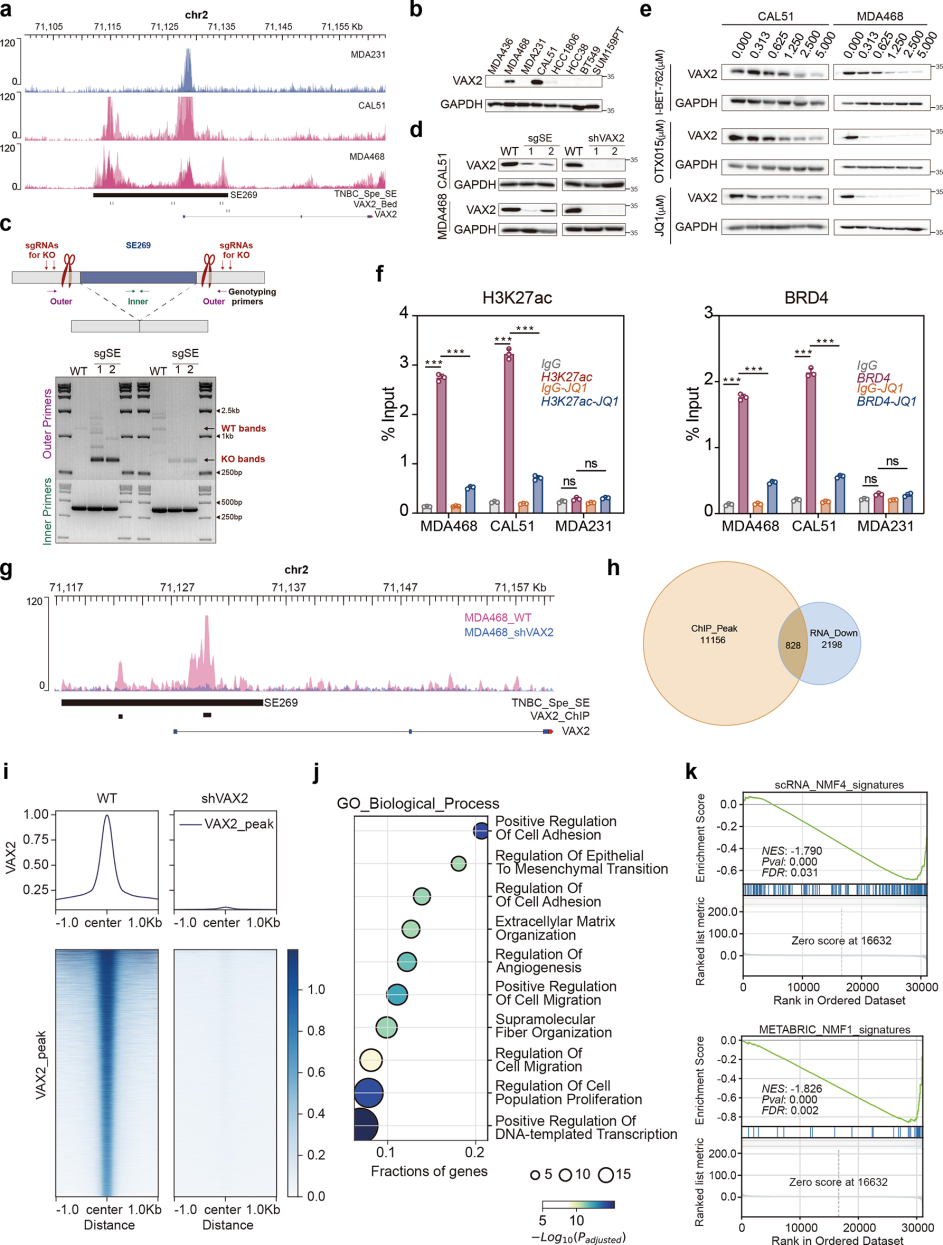
VAX2 is a key master regulator for the TNBC mesenchymal development subtype. (**a**) Genome browser plot showing TNBC-specific SE and key master regulator VAX2 H3K27ac signals. The data were obtained from public databases, refer to Supplementary Table 1 for details. (**b**) Immunoblotting detection of VAX2 expression in a panel of TNBC cell lines. (**c**) Top: Schematic illustrating CRISPR-knockout of SE269. Bottom: DNA blot confirming successful heterozygous knockout of SE269. (**d**) Immunoblotting of VAX2 in CAL51 and MDA468 upon deletion of SE269 or direct knockdown of VAX2. (**e**) Inhibition of VAX2 expression levels in CAL51 and MDA468 cell lines by BETi, with the inhibitory effect increasing as the drug concentration increases. (**f**) H3K27ac and BRD4 ChIP-qPCR of the indicated cell lines using primers amplifying VAX2 SE269. Error bars represent mean ± SD, n = 3 biological independent samples. (**g**) IGV map showing VAX2 peaks and VAX2 ChIP-seq binding locations around SE269 in MDA468 and VAX2-knockdown MDA468 cell lines. (**h**) ChIP-seq analysis of VAX2 identified 11,156 potential target genes. Among them, 828 genes were downregulated in VAX2-knockdown cells. (**i**) ChIP-seq signal heatmap showing that VAX2 is enriched in 828 regulated genes. (**j**) Functional enrichment analysis of 828 genes regulated by VAX2, identified by VAX2 ChIP-seq and RNA-seq, showed relevance to mesenchymal development functions (including EMT, ECM formation, cell proliferation, cell adhesion, etc.). (**k**) GSEA of differentially expressed genes between VAX2-knockdown and wild-type MDA468 cell lines showed attenuated enrichment of gene sets characteristic of the TNBC mesenchymal development subtype. The *p* value in (**f**) was determined by a two-sided Student's t-test. *** *p* < 0.001; ns, no significance. Data in (**b–f**) were representative of three independent experiments.

To investigate the biological function of the master regulator VAX2 in the mesenchymal development subtype of TNBC, we first selected appropriate cell lines for experimental validation. Based on previous analyses, the MDA468 and CAL51 cell lines were identified as representative of this subtype (Fig. 2a). H3K27ac ChIP-seq data revealed significant TNBC-specific SE coverage near the VAX2 gene in these cell lines (Fig. 6a). Additionally, VAX2 expression was markedly higher in MDA468 and CAL51 compared to other TNBC cell lines (Fig. 6b). In contrast, the MDA231 cell line, which does not belong to the mesenchymal development subtype, exhibited negligible SE coverage near the VAX2 gene and had very low VAX2 expression (Fig. 6a and b). Therefore, we selected MDA468, CAL51, and MDA231 for subsequent functional validation.

We conducted a series of experiments to verify that VAX2, as a master regulator of the mesenchymal development subtype, is regulated by TNBC-specific SE. Using CRISPR/Cas9, we excised proximal and distal regions of SE269 (chr2: 71,112,006–71,135,503) in MDA468 and CAL51 cells to examine its regulatory effect on VAX2 expression (Fig. 6c). This excision led to a significant reduction in VAX2 protein levels, mirroring the effect of direct VAX2 knockdown (Fig. 6d). Considering that SEs are enriched with H3K27ac marks and the transcriptional cofactor BRD4, we treated with the BETi JQ1 to disrupt BRD4 recruitment to the SE, thereby disrupting its function. Increasing JQ1 concentrations resulted in a notable decrease in VAX2 protein levels in MDA468 and CAL51 cells (Fig. 6e). Subsequent ChIP-qPCR analysis demonstrated that SE269 was enriched with H3K27ac and BRD4 in MDA468 and CAL51, but not in MDA231 cells. After 48 h of JQ1 treatment, the enrichment of H3K27ac and BRD4 at SE269 was significantly reduced in MDA468 and CAL51 cells (Fig. 6f). Collectively, our experiments confirm that elevated VAX2 expression in the mesenchymal development subtype is driven by TNBC-specific SE.

Given that VAX2 serves as the master regulator of the mesenchymal development subtype, we performed ChIP-seq and RNA-seq analyses in wild-type and VAX2-knockdown MDA468 cell lines to elucidate its regulatory mechanism. The results revealed that VAX2 binds to the SE269, suggesting that SE269 not only regulates VAX2 expression but that VAX2, in turn, binds again to SE269 to promote its own expression, establishing a transcriptional CRC between TNBC-specific SE and VAX2 (Fig. 6g). This mechanism likely plays a pivotal role in the mesenchymal development TNBC subtype. ChIP-seq analysis identified 11,156 potential target genes, among which 828 exhibited a significant decrease in ChIP-seq signal upon VAX2 knockdown, accompanied by a simultaneous downregulation in RNA expression levels, indicating that the expression regulation of these genes is closely related to VAX2 (Fig. 6h and Supplementary Table 11). Furthermore, ChIP-seq signal heatmaps demonstrated a reduction in signal intensity near these genes following VAX2 knockdown, further confirming its regulatory role (Fig. 6i). Functional enrichment analysis showed that these 828 genes were significantly enriched in mesenchymal development processes, including cell adhesion, migration, proliferation, EMT, and ECM remodeling, suggesting that VAX2 influences the characteristics of the mesenchymal development subtype through their regulation (Fig. 6j).

Further analysis revealed that VAX2 knockdown led to decreased ChIP-seq signals and RNA-seq expressions for key molecules of the RHO pathway (RAC1, RAC3), EMT-associated mesenchymal markers (FN1, MMP9, TGFBI), and IL6, a critical factor in CAF maturation (Supplementary Fig. 6a-h). These regulatory relationships were further validated by qPCR, supporting the central role of VAX2 in the mesenchymal development subtype (Supplementary Fig. 6i). Simultaneously, VAX2 knockdown reduced the enrichment of the mesenchymal development subtype signature genesets, indicating its essential role in maintaining the subtype characteristics (Fig. 6k). Additionally, RNA-seq analysis showed a significant downregulation of ECM organization and collagen synthesis pathways upon VAX2 knockdown, both of which are highly enriched in the mesenchymal development subtype (Supplementary Fig. 6j). Collectively, our findings reveal that VAX2 establishes a feedforward regulatory loop with TNBC-specific SEs to enhance its own expression and amplify its downstream transcriptional network, thereby playing a crucial role in the maintenance and regulation of the mesenchymal development subtype of TNBC.

### Mesenchymal development subtype TNBC driven by VAX2 exhibits high malignancy and is susceptible to BETi

**Fig. 7:**
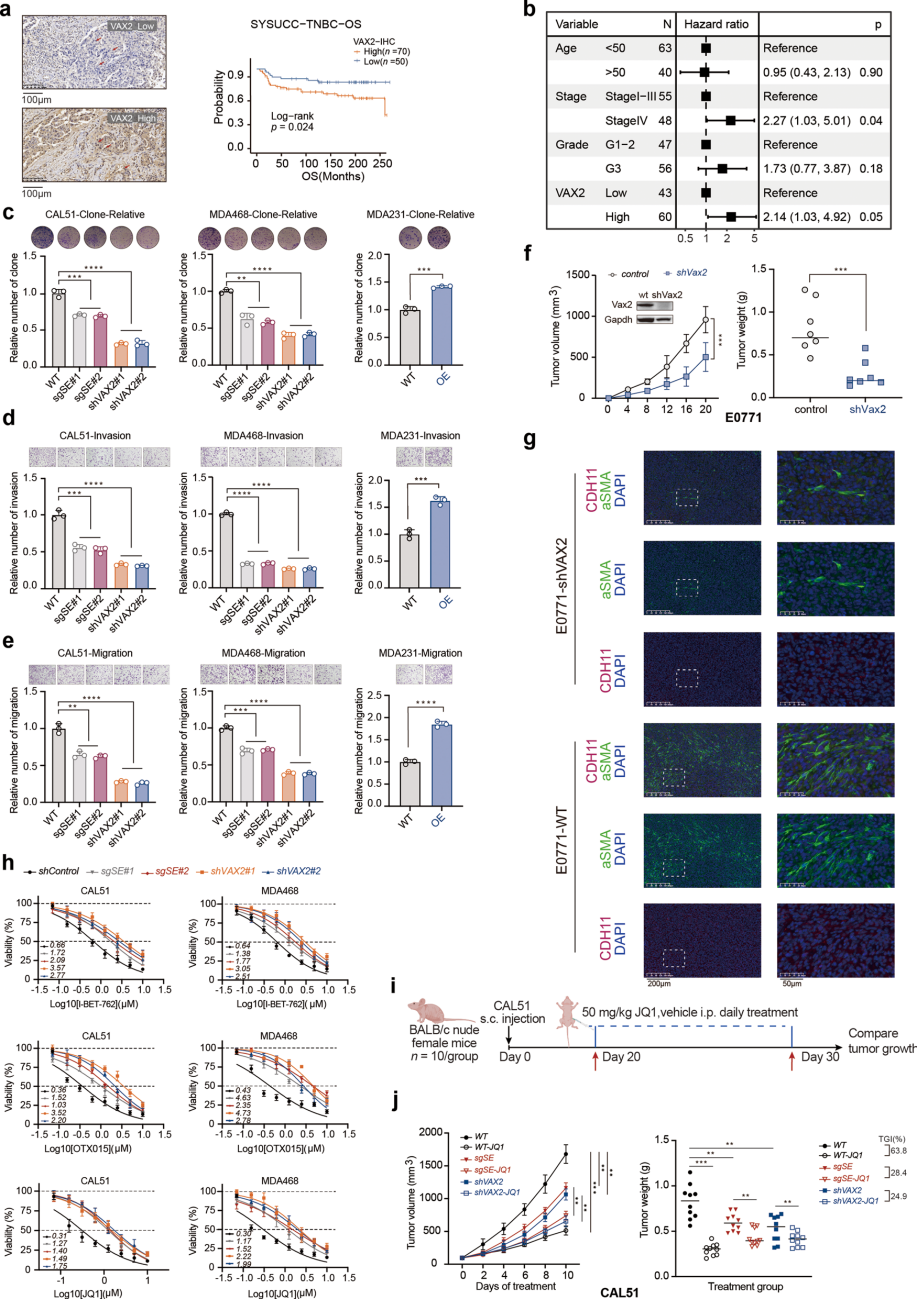
The VAX2-driven mesenchymal development subtype of TNBC exhibits high malignancy and heightened sensitivity to BETi. (**a**) Clinical data from SYSUCC-TNBC analysis on the impact of VAX2 immunohistochemical staining intensity on patient prognosis. (**b**) Multivariate Cox model analysis of the effect of VAX2 immunohistochemical staining intensity on OS in SYSUCC-TNBC. (**c–e**) Effects of VAX2 SE269 excision, direct VAX2 knockdown in CAL51 and MDA468 cell lines, and VAX2 overexpression in MDA231 cells on cell proliferation (**c**), migration (**d**), and invasion (**e**). n = 3 biological independent samples. (**f**) Tumour growth of the indicated E0771 cells in C57BL/6 mice (n = 7 mice per group). (**g**) Multiple immunofluorescence analyses showing the effect of VAX2 expression on mCAF infiltration. (**h**) Sensitivity of wild-type TNBC mesenchymal development subtype cell lines, SE269-deleted lines, and VAX2-knockdown lines to BETi, n = 6 biological independent samples. (**i-j**) Schematic of JQ1 in vivo therapy experiments (n = 10 mice per group). Blue dashed lines indicate the JQ1 dosing period. (**i**). Tumor growth curves and weight measurements of CAL51 and modified strains from BALB/c nude mice 30 days after vehicle or JQ1 treatment (**j**). Error bars represent mean ± SD. The *p* value in (**c**, **d**, **e**) was caluculated by a two-sided Student's t-test. The *p* value in (**f**) and (**j**) was determined by one-way ANOVA, ** *p* < 0.01, *** *p* < 0.001. Data in (**c, d, e, h**) were representative of three independent experiments. Data in **(f, g, i, j**) were representative of two independent experiments.

To evaluate the clinical relevance of VAX2 in TNBC, we analyzed its staining intensities in patient samples by immunohistochemistry and assessed its association with survival prognosis. Patients were stratified into high and low VAX2 staining-intensity groups based on immunohistochemistry results from an analysis of 120 TNBC clinical samples (Fig. 7a and Supplementary Table 4). Higher VAX2 staining intensities were significantly correlated with poorer OS (Fig. 7a). Univariate Cox regression analysis revealed a hazard ratio of 2.30 for OS, while multivariate Cox regression analysis, adjusted for age, tumor stage, and grade, confirmed VAX2 as an independent risk factor for OS, with a hazard ratio of 2.14 (Fig. 7b). These findings suggest that VAX2 contributes to tumor malignancy and serve as a prognostic biomarker for poor patient outcomes.

To further investigate the role of VAX2 in tumor malignancy, we conducted both in vitro and in vivo experiments. In vitro, ablation of SE269 or knockdown of VAX2 in MDA468 and CAL51 cells significantly suppressed tumor cell proliferation, invasion, and migration. Conversely, VAX2 overexpression in MDA231 cells enhanced these malignant phenotypes (Fig. 7c-e). In human xenograft models in vivo, VAX2 overexpression in MDA231 cells accelerated tumor growth and increased tumor burden in BALB/c nude mice (Supplementary Fig. 7a). Additional experiments demonstrated that VAX2 knockdown markedly inhibited tumor growth in E0771 and 4T1 cell-derived xenograft models and substantially reduced tumor burden in C57BL/6 and BALB/c mice (Fig. 7f and Supplementary Fig. 7b-c). As previous analyses revealed an association between VAX2 and the mesenchymal development features, as well as its potential involvement in ECM remodeling, we further examined its specific effects on the ECM. Multiplex immunofluorescence analysis of E0771 mouse tumor tissues showed that VAX2 knockdown significantly decreased the number of mCAFs within the tumor microenvironment (Fig. 7g and Supplementary Fig. 7d). These findings suggest that VAX2 plays a crucial role in promoting TNBC progression by modulating mCAF abundance and function to influence ECM remodeling.

As we demonstrated that the CRC formed by SE269-VAX2 amplified the transcriptional regulatory network of VAX2, we hypothesized that BETi might interfere with this feedforward regulatory loop by suppressing SE function. To test this hypothesis, we conducted IC50 assays to assess the sensitivity of the TNBC mesenchymal subtype to three BETis: I-BET-762, OTX015, and JQ1. These experiments were performed on MDA468 and CAL51 cell lines, including modified strains with SE269 disrupted or VAX2 knocked down. Our results indicated that disrupting SE269 or knocking down VAX2 expression reduced sensitivity to BETi (Fig. 7h). Specifically, in CAL51 cells, the IC50 values for I-BET-762, OTX015, and JQ1 were 0.66 µM, 0.36 µM, and 0.31 µM, respectively, while in MDA468 cells, these values were 0.64 µM, 0.43 µM, and 0.30 µM, respectively. Wild-type cell lines exhibited greater sensitivity to BETi than the modified strains (Fig. 7h). To further investigate the effects of BETi in vivo, we implanted CAL51 cell lines, as well as CAL51 strains with disrupted SE269 or VAX2 knockdown, into BALB/c nude mice. After twenty days, the tumors were treated with JQ1 at 50 mg/kg for 10 days (Fig. 7i). Actually, tumors derived from CAL51 wild-type cells exhibited more rapid growth and a larger tumor burden compared to those derived from the modified strains (Fig. 7j). Additionally, the CAL51 wild-type tumors responded more significantly to BETi treatment, with a tumor growth inhibition (TGI) rate of 63.8%. In contrast, tumors lacking complete SE269 or with reduced VAX2 expression had TGI rates of 28.4% and 24.9%, respectively (Fig. 7j). No obvious toxicity was observed in the mice receiving the treatment. Together, these data indicate that the mesenchymal development subtype of TNBC driven by VAX2 are sensitive to BETi.

### Development and validation of a predictive model for mesenchymal development subtype in TNBC

**Fig. 8:**
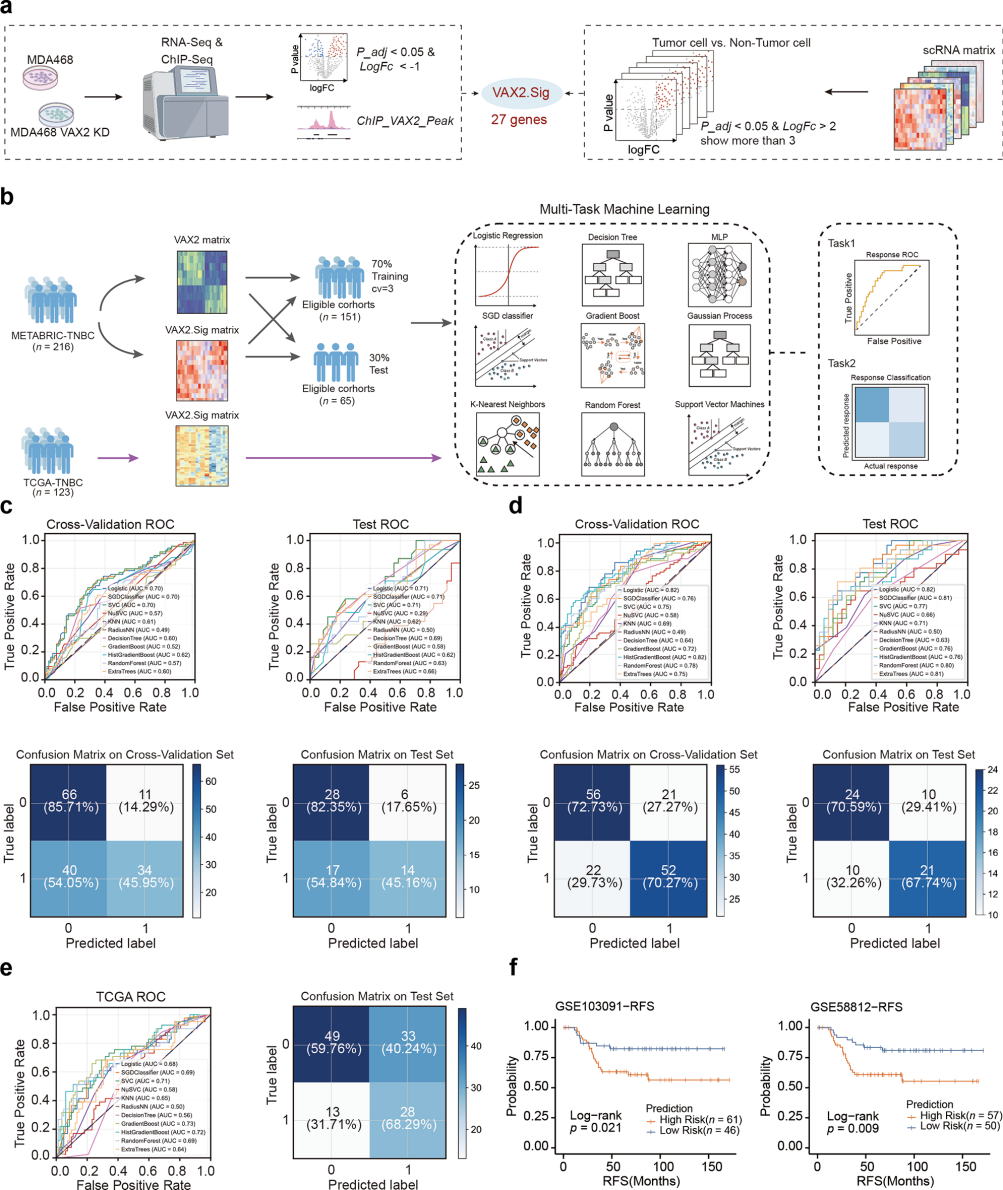
A simple machine-learning model predicts TNBC mesenchymal subtype patients. (**a**) Flowchart illustrating the construction of the VAX2 signature (VAX2.sig). First, downregulated genes (p.adj < 0.05 & logFC < -1) were identified from RNA-seq of VAX2-knockdown MDA468 vs. WT-MDA468. Second, genes near VAX2-binding peak regions were obtained from ChIP-seq analysis of VAX2-knockdown MDA468 vs. WT-MDA468. Finally, genes specifically expressed in tumor cells were identified from six TNBC single-cell datasets (p.adj < 0.05 & logFC > 2; genes present in more than three datasets). The intersection of these gene sets was taken to define VAX2.sig. (**b**) VAX2 or VAX2.sig expression was used in a multi-task machine-learning model to predict patients with the TNBC mesenchymal subtype. METABRIC-TNBC was used for model training and validation, and TCGA-TNBC was used for independent validation. (**c-d**) In METABRIC-TNBC, the predictive performance of different machine-learning models based on VAX2 expression (**c**) or VAX2.sig (**d**), including the ROC curves of different machine-learning models and the confusion matrix of the best-performing model. (**e**) In TCGA-TNBC, the predictive performance of different machine-learning models based on VAX2.sig, including the ROC curves of different machine-learning models and the confusion matrix of the best-performing model. (**f**) The best-performing machine-learning model based on VAX2.sig predicted the prognosis of patients with the mesenchymal subtype in two additional TNBC cohorts.

Identifying the mesenchymal development subtype of TNBC using H3K27ac ChIP-seq and transcriptome sequencing data is technically challenging and resource-intensive. Therefore, developing a streamlined yet robust predictive model for this TNBC subtype would provide significant benefits for clinical and translational applications. We constructed a predictive model by integrating large-scale analysis with multi-task machine learning (Supplementary Table 5). Initially, genes regulated by VAX2 were identified through ChIP-seq and RNA-seq analysis of the MDA468 cell line. Next, tumor cell-specific highly expressed marker genes were determined from TNBC scRNA-Seq data, selecting genes specifically expressed in tumor cell in at least four datasets. The intersection of these two gene sets yielded 27 genes regulated by VAX2 and highly expressed specifically in tumor cells, forming the VAX2 signature (VAX2.Sig) (Fig. 8a).

In the METABRIC study, using VAX2 expression alone for supervised learning to predict the mesenchymal development subtype of TNBC demonstrated limited effectiveness, with a maximum AUC of 0.7 in both the training and validation sets (Fig. 8b and c). To enhance prediction performance, we utilized the expression profile of the VAX2.Sig for supervised learning. In the training and test sets, various models—including Logistic Regression, SGDClassifier, SVC, HighGradientBoost, RandomForest, and ExtraTrees—all achieved AUCs greater than 0.75 (Fig. 8b and d). The model was further validated using an independent TCGA-TNBC cohort, where the AUCs for SVC and HighGradientBoost models were all greater than or equal to 0.7. Between these two models, HighGradientBoost demonstrated the better predictive performance in the independent validation set (Fig. 8b and e).

To evaluate the practical applicability of the HighGradientBoost best-performing model, we predicted TNBC the mesenchymal development subtypes in two additional cohorts, GSE103091-TNBC and GSE58812-TNBC. The results showed that patients predicted to have the mesenchymal development subtype of TNBC had shorter RFS (Fig. 8f). Furthermore, the mean expression level of the VAX2 gene was higher in patients predicted to have the mesenchymal development subtype compared to those with the non-mesenchymal subtype (Supplementary Fig. 7e). Additionally, patients predicted to have the mesenchymal development subtype showed significant enrichment in the mesenchymal development subtype signature gene set (Supplementary Fig. 7f and g). Taken together, our study proposes a simple and effective model for predicting the mesenchymal development subtypes of TNBC using a VAX2-based gene signature.

## Discussion

The high heterogeneity of tumors currently limits the efficacy of BETi in targeting solid tumors, including TNBC, in clinical trials. Our study demonstrates that a heterogeneity analysis of TNBC-specific SEs identified a distinct mesenchymal development subtype characterized by high malignancy and increased susceptibility to BETi. The SE landscape of TNBC is distinct from that of non-TNBC and normal mammary epithelium. These TNBC-specific SEs contribute to tumor heterogeneity, enabling the classification of TNBC into functional subtypes. We identified a consistently present mesenchymal development subtype characterized by elevated clinical malignancy and an ECM-enriched TME. This high-risk subtype is driven by a transcriptional CRC composed of TNBC-specific SEs and the master regulator VAX2. Disrupting the SEs regulating VAX2 or knocking down VAX2 expression significantly reduced the malignant characteristics of this subtype, leading to the downregulation of ECM-related pathways and the signature gene sets of the mesenchymal development subtype. Moreover, the transcriptional CRC further increased the sensitivity of the mesenchymal development subtype cells to BETi, which targets SE function. Given VAX2’s role as a master regulator of the mesenchymal development subtype, we developed a machine learning model based on the VAX2 regulatory network to accurately predict this high-risk subtype. This model is intended to facilitate precision therapy by identifying patients who may benefit most from BETi treatment.

The mesenchymal development subtype of TNBC is characterized by highly malignant clinical features, closely linked to the hybrid EMT status of tumor cells and an ECM-enriched TME. Recent advances in cell fate lineage tracing have highlighted the diversity and heterogeneity of EMT states, categorizing them into partial EMT, intermediate EMT, extreme EMT, and amoeboid EMT states [47]. Among these, partial and intermediate EMT states, collectively referred to as hybrid EMT, are more invasive than other EMT states [47]. Tumors in hybrid EMT states exhibit enhanced proliferation, invasion, metastatic potential, and stem cell-like properties [47]. Additionally, this mesenchymal development subtype shows increased ECM-enrichment and a closer spatial association with mCAFs compared to other subtypes. Numerous studies have demonstrated that ECM and mCAFs can promote tumor progression both directly and indirectly, contributing to tumor malignant progression [48, 49]. mCAFs not only secrete numerous cytokines, including TGFβ, HGF, IL-6, CCL5, and MMP9, activating TGF-β, IL, and NF-κB signaling pathways [50], and inducing EMT in tumor cells, but also remodel the ECM by regulating the production of collagen and laminin, leading to the chronic accumulation of ECM proteins and the formation of a dense, high-pressure environment [51]. This ECM remodeling results in vascular collapse, impeding the transport of oxygen and nutrients, and creating a hypoxic and nutrient-deficient TME [51]. Our results demonstrated that the mesenchymal development subtype of TNBC exhibited elevated expression of both epithelial and mesenchymal marker genes, indicative of a hybrid EMT state. Compared to the non-mesenchymal development subtypes, this subtype displayed enhanced activity of ECM-related pathways. Furthermore, spatial analysis of the TME revealed a closer association with mCAFs, which actively contributed to ECM remodeling and formation. These findings underscore the critical role of the hybrid EMT state and ECM-enriched TME in driving the malignant progression of the mesenchymal development subtype.

The transcriptional CRC formed by SEs and the master regulator is crucial in determining tumor cell fate. In this study, we combined bioinformatics analyses with experimental validation to establish that VAX2, regulated by SEs in the mesenchymal development subtype, is a key driver of the subtype’s formation and progression. Research on VAX2 in cancer is relatively limited, and its role seems to vary across different malignancies. For example, in high-grade non-muscle-invasive bladder cancer, VAX2 functions as a tumor suppressor, as evidenced by increased methylation and decreased expression in high-grade tumors [52]. In contrast, in papillary thyroid carcinoma, elevated VAX2 expression is associated with poor prognosis, primarily due to the activation of the MEK/ERK signaling pathway, which promotes tumor cell proliferation, migration, and invasion [53]. Similarly, VAX2 is significantly overexpressed in gastric cancer tissues and is correlated with advanced-stage disease and positive lymph node metastasis [54]. Functional studies have shown that high VAX2 expression significantly enhances the proliferation and invasion of gastric cancer cells. Our study further demonstrated that VAX2 played an oncogenic role in the mesenchymal development subtype. We found that VAX2 drived the expression of characteristics of this subtype, activated ECM-related pathways, and directly promoted tumor cell proliferation, invasion, and migration. These findings provide a scientific foundation for developing targeted therapies against this high-risk TNBC subtype.

The limited clinical efficacy of BETi is likely influenced by tumor heterogeneity [37]. In normal tissues, genes regulated by SEs are cell- and tissue-specific and are involved in diverse biological processes such as metabolism, immunity, proliferation, and migration [13]. Similarly, in tumor tissues, SE-regulated biological processes exhibit significant heterogeneity. For example, colon cancer can be classified into subtypes (Epic1, Epic2, Epic3, Epic4) based on enhancer and SE landscapes, with the Epic1 subtype characterized by genes related to immune regulation and inflammatory response [18]. Furthermore, SEs not only promote oncogene expression and tumor progression but also regulate tumor suppressor genes like RCAN1.4, which inhibits tumorigenesis [55]. Therefore, the heterogeneity of SEs in tumors suggests that the indiscriminate use of BETi could compromise SE-regulated immune functions and inhibit the expression of tumor suppressor genes, complicating effective tumor control. Our study identified significant SE heterogeneity in TNBC, particularly highlighting a high-risk mesenchymal development subtype driven by transcriptional CRCs involving TNBC-specific SEs and the master regulator VAX2. This subtype exhibited heightened sensitivity to BETi. Disruption of the CRC by deleting TNBC-specific SEs or downregulating VAX2 expression attenuated the malignant characteristics of this mesenchymal subtype and reduced its sensitivity to BETi. To translate these findings into clinical practice, we developed a multi-task machine-learning model based on the VAX2 regulatory network, which is capable of accurately identifying the mesenchymal development subtype of TNBC and guiding the selection of targeted therapies.

## Conclusions

In conclusion, our study underscores the critical role of SEs in TNBC heterogeneity and their potential to guide targeted BETi treatments. By integrating epigenomic, transcriptomic, and single-cell data, we comprehensively analyzed the SE landscape in TNBC, revealing the heterogeneity of TNBC-specific SEs. We identified a mesenchymal development subtype regulated by a transcriptional CRC formed by SEs and the master regulator VAX2, which exhibits heightened sensitivity to BETis. These findings will contribute to the precision treatment of TNBC.

## Electronic supplementary material

Below is the link to the electronic supplementary material.

Supplementary Material 1

Supplementary Material 2

## Data Availability

All data generated or analyzed during this study are included in the supplementary files. Public datasets were obtained from GEO, TCGA, cBioPortal, and the DepMap database, with corresponding data accession numbers detailed in the Methods section. All original analysis codes have been deposited in Figshare at 10.6084/m9.figshare.28669214.v1 and are publicly available.
