# Supplementary material for: Profiling triple-negative breast cancer-specific super-enhancers identifies high-risk mesenchymal development subtype and BETi-Targetable vulnerabilities
Source: Mol Cancer. 2025 May 13;24:141. doi: 10.1186/s12943-025-02342-6 (PMC12070678; doi:10.1186/s12943-025-02342-6)
Supplement: Supplementary file 1 — Supplementary Material 1 [file 12943_2025_2342_MOESM1_ESM.docx]

Supplementary Materials for

**Profiling Triple-negative breast cancer-specific Super-Enhancers Identifies High-Risk Mesenchymal Development Subtype and BETi-Targetable Vulnerabilities**

Qing-shan Chen^1,2†^, Rui-zhao Cai^1,2†^, Yan Wang^1,2†^, Ge-hao Liang^1,2^, Kai-ming Zhang^1,2^, Xiao-Yu Yang^1^, Dong Yang^1^, De-Chang Zhao^1,2^, Xiao-Feng Zhu^1*^, Rong Deng^1*^, Jun Tang^1,2*^

^1^State Key Laboratory of Oncology in South China, Guangdong Provincial Clinical Research Center for Cancer, Sun Yat-sen University Cancer Center, Guangzhou 510060, P. R. China

^2^Department of Breast Oncology, Sun Yat-sen University Cancer Center, Guangzhou, China

Correspondence to: [tangjun@sysucc.org](mailto:tangjun@sysucc.org).cn

**This PDF file includes:**

Supplementary Figures. S1 to S5

**
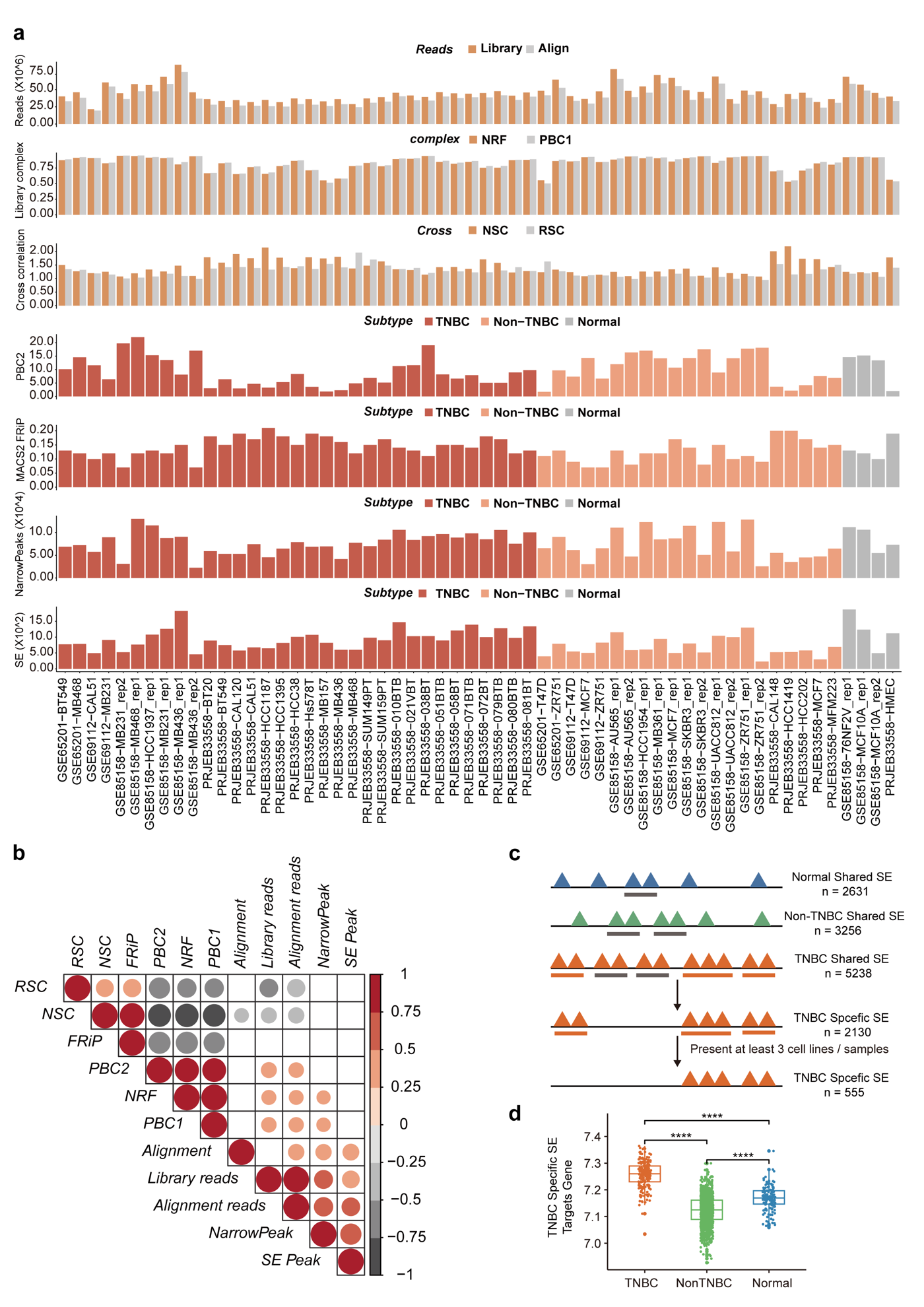
**

**Supplementary Fig. 1 The H3K27ac ChIP-seq analysis in breast cancer and normal mammary epithelium. a** Quality parameter for the H3K27ac ChIP-seq analysis in TNBC patients (n = 10), TNBC cell lines (n = 15), non-TNBC cell lines (n = 11), and normal mammary epithelium (n =3), including the number of aligned reads; Non-Redundant Fraction (NRF) and PCR Bottlenecking Coefficients 1 and 2 (PBC1 and PBC2), normalized strand correlation (NSC), relative strand correlation (RSC) as defined by ENCODE; fraction of reads in MACS2 peaks (FRiP); number of enhancer peaks called by MACS2; number of SE peaks called by ROSE. **b** Pairwise Spearman correlation between all of the QC parameters shown in (**a**). **c** Comparative analysis of the scheme to obtain TNBC-specific SEs. **d** Boxplot representing the mean expression of TNBC-specific SE target genes in TNBC, non-TNBC, and normal mammary epithelium from METABRIC. The centerline depicts the median values; the bottom and top box edges correspond to the first and third quartiles. Statistical analysis was performed using the Wilcoxon rank-sum test. **** *p* < 0.0001.

**
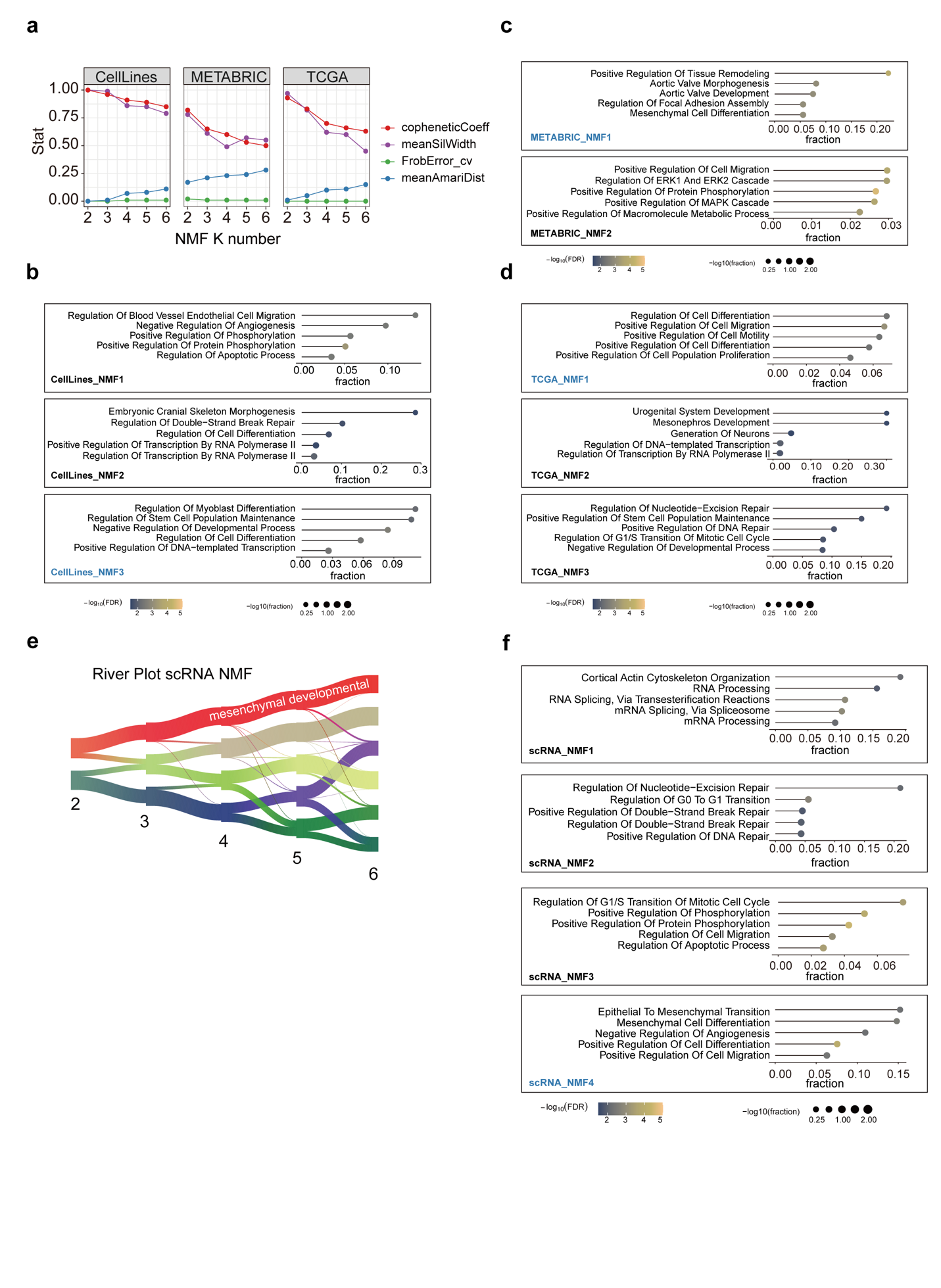
**

**Supplementary Fig. 2 Heterogeneous subtypes defined by TNBC-specific SE. a** Evaluation indicators of typing effect of different numbers of NMF typing. The optimal number of NMF typing is when the copheneticCoeff and meanSilWidth curves drop the most, and FrobError_cv and meanAmariDist are small. **b-d** Biological functions of characteristic genes of different NMF types of TNBC cell lines (**b**) and TNBC patients from METABRIC (**c**) and TCGA (**d**). **e** River-plot representation of the stability of the signatures extracted from the TNBC-specific SE targets in tumor cells. The horizontal axis represents the different factorization ranks (which equals to the number of signatures extracted, k=2 to 6) and the ribbons indicate the similarity of the signatures defined for different factorization ranks. **f** Biological functions of characteristic genes of different NMF types of TNBC tumor cells from scRNA-Seq.

**
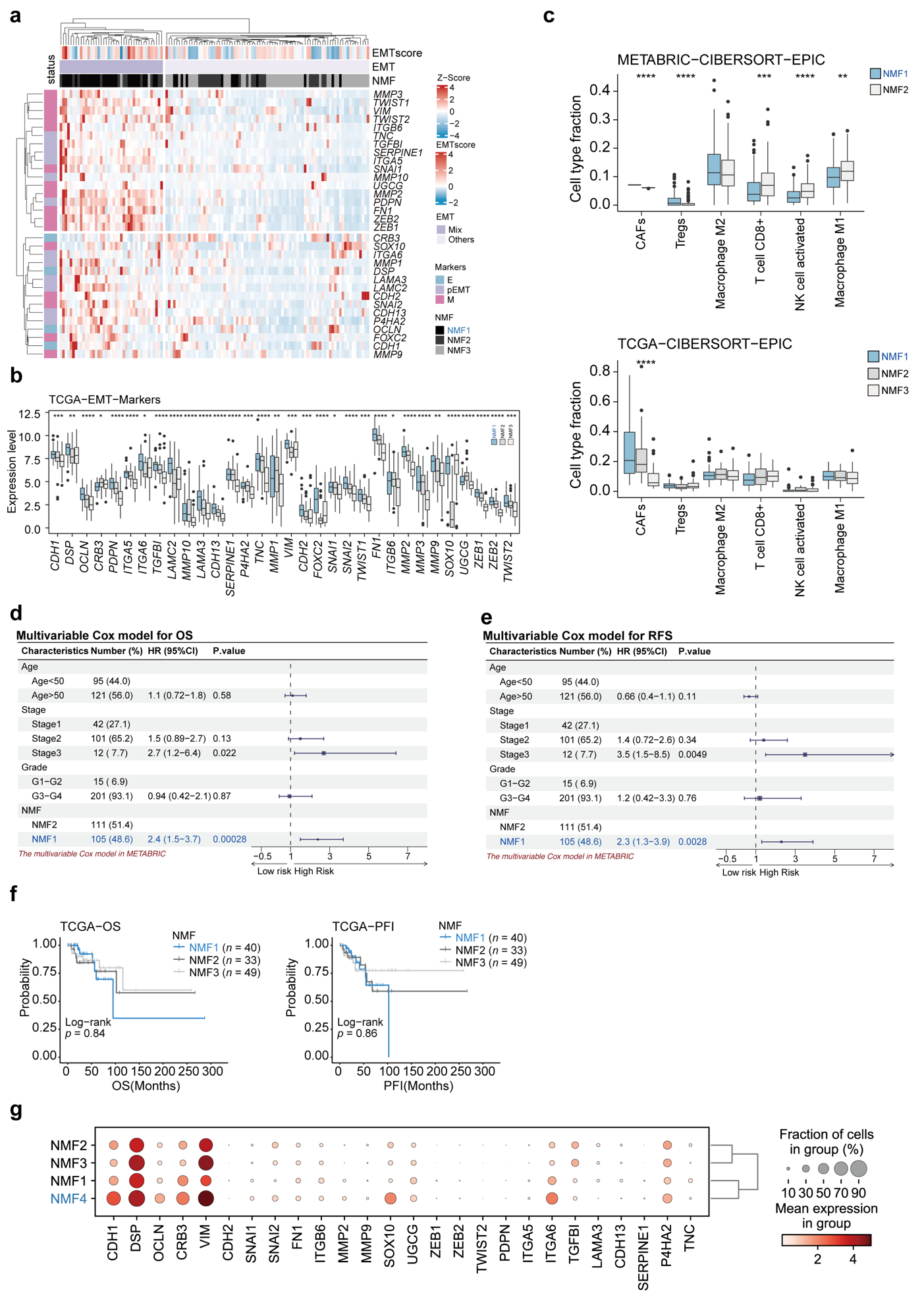
**

**Supplementary Fig. 3 Biological characteristics of heterogeneous subtypes defined by TNBC-specific SE.** **a-b** EMT scores and status (**a**), mean expression level of EMT marker genes (**b**) in different TNBC NMF subtypes from TCGA. **c** Infiltration of CAFs and immune cells in different NMF subtypes of TNBC from METABRIC and TCGA. **d-e** Multivariate Cox model analysis of the impact of NMF subtype on OS (**d**) and RFS (**e**) from METABRIC-TNBC. **f** Kaplan–Meier OS and progression-free interval (PFI) curves for patients assigned to different NMF subtypes in TCGA-TNBC. **g** Dotplot shows the expression of EMT marker genes in different NMF subtypes of TNBC tumor cells. Statistical analysis was performed using Wilcoxon rank-sum test. * *p* < 0.05, ** *p* < 0.01, *** *p* < 0.001, **** *p* < 0.0001.

**
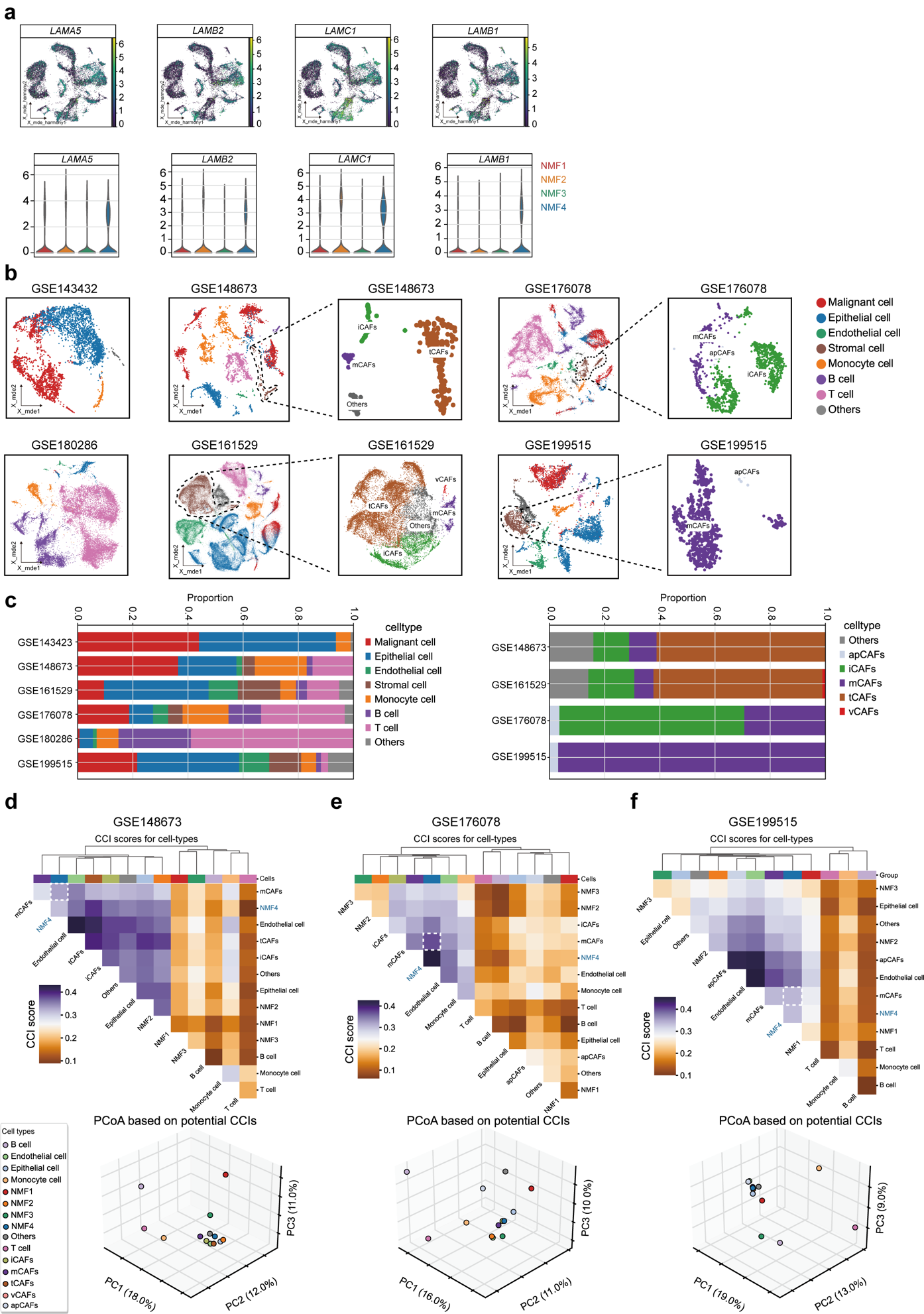
**

**Supplementary Fig. 4 The tumor microenvironment for TNBC mesenchymal developmental subtypes. a** BM component expression level in different subtypes from TNBC scRNA-seq dataset. **b-c** Cell type annotation (**b**), composition and proportion (**c**) in different TNBC scRNA-Seq datasets. **d-f** cell-cell communication strength between tumor cells of different NMF subtypes and mCAFs and estimated spatial Euclidean distances from multiple TNBC scRNA-Seq datasets.


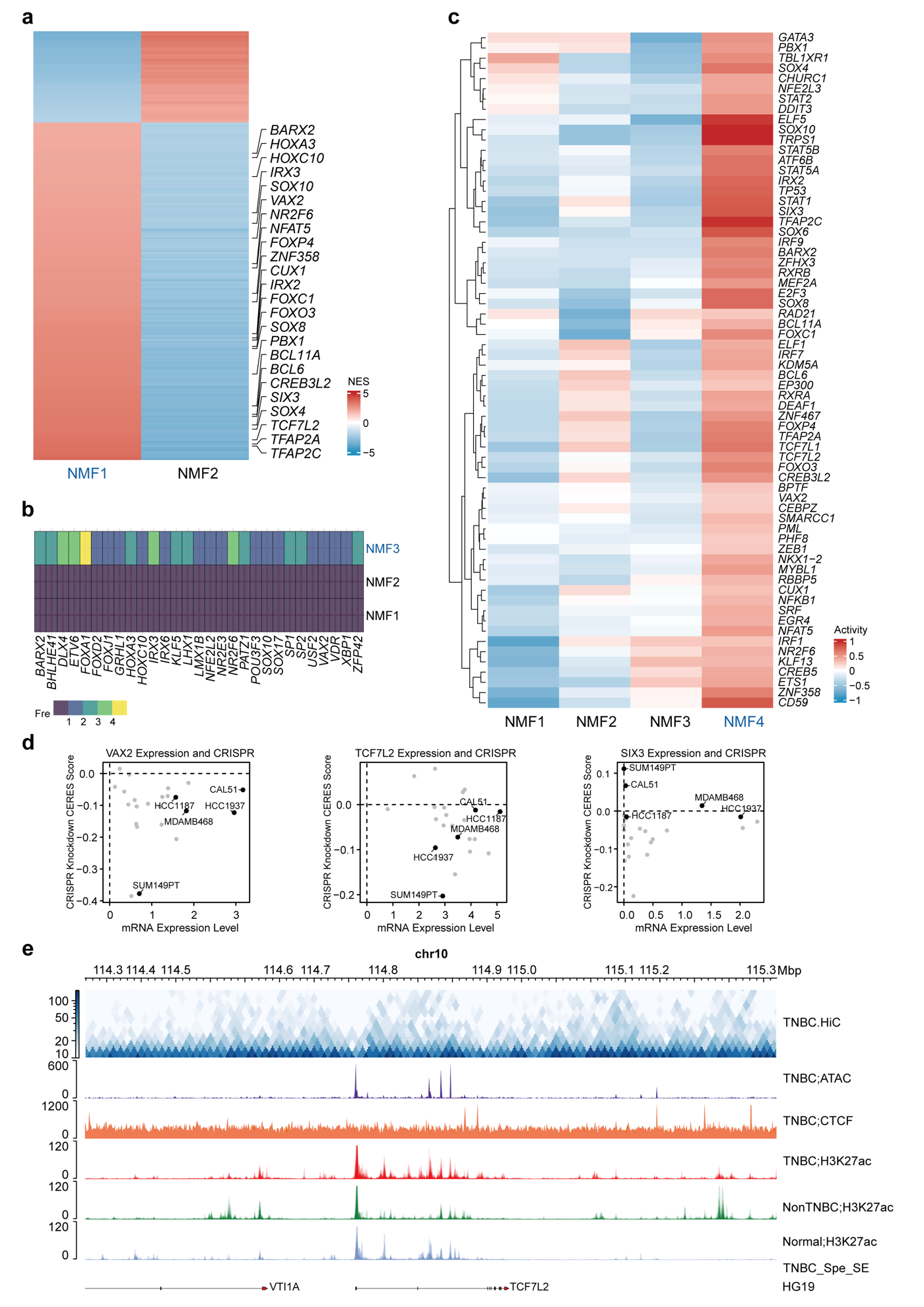


**Supplementary Fig. 5 The master regulator for TNBC mesenchymal developmental subtypes. a-c** the core TF and compute the TF activities based on the ARACNe-AP and msVIPER methods (**a**), CRCmapper (**b**) and pySCENIC algorithm (**c**). **d** Expression of transcription factors VAX2, TCF7L2 and SIX3 in TNBC cell lines and their effects on cell survival. The indicated cell lines are TNBC mesenchymal subtypes. **e** Integration of multiple layers of regulatory information, including Hi-C, CTCF, ATAC-seq, and H3K27ac ChIP-seq profiles, TNBC-specific SEs, and chromatin interactions, exemplified by the TCF7L2 locus.


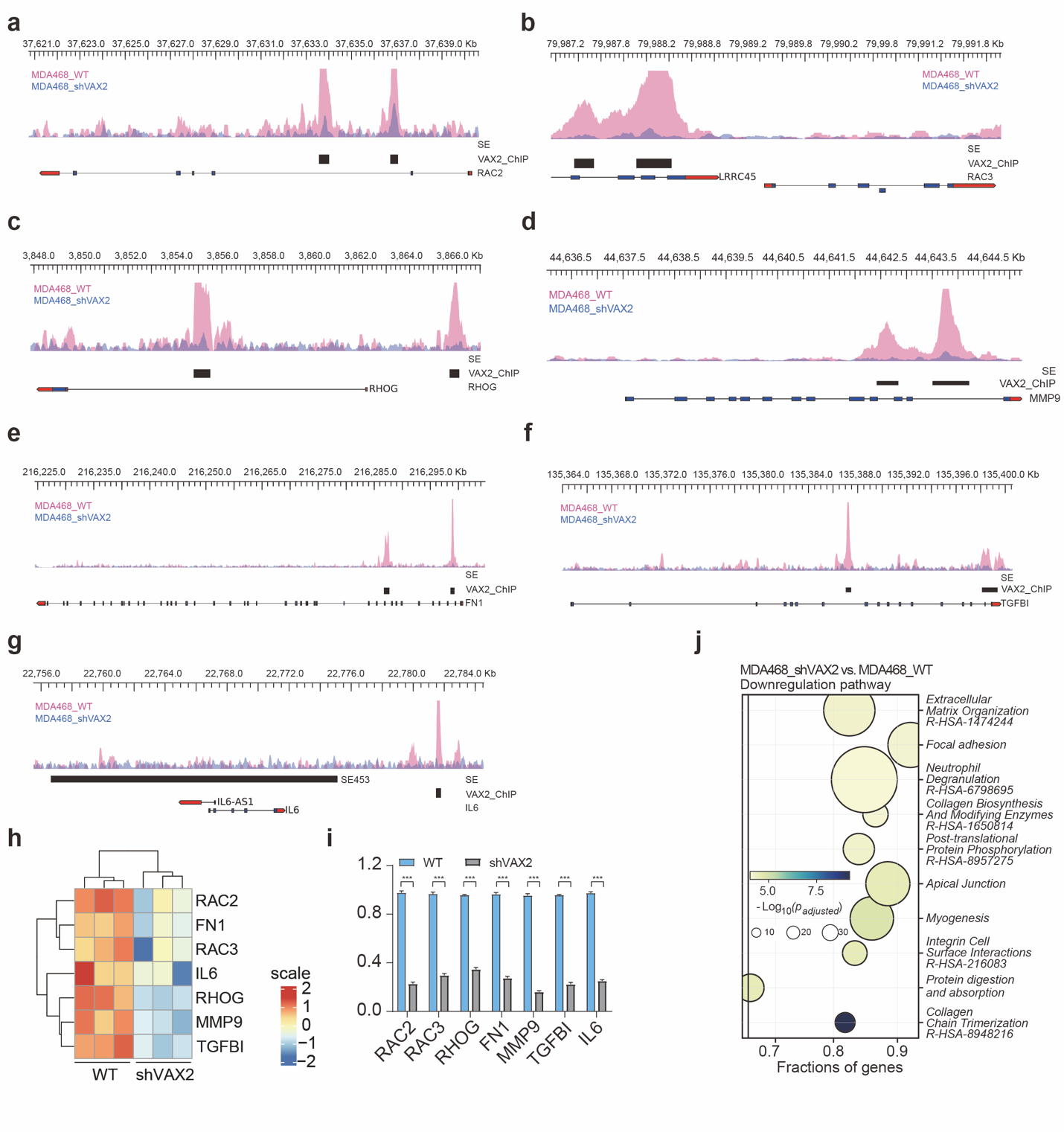


**Supplementary Fig. 6 VAX2 is a key master regulator for the TNBC mesenchymal development subtype. a-g** IGV tracks showing VAX2-binding peaks and ChIP-seq binding sites around RHO pathway-related molecules (RAC2, RAC3, RHOG), EMT marker genes (FN1, MMP9, TGFBI), and IL6 in MDA468 and VAX2-knockdown MDA468 cell lines. **h** Heatmap showing the expression of RHO pathway-related molecules, EMT marker genes, and IL6 in VAX2-knockdown and wild-type MDA468 cells based on RNA-seq. **i** qPCR validation further confirmed the decreased expression of RHO pathway-related molecules, EMT marker genes, and IL6 in VAX2-knockdown cells. Error bars represent mean ± SD, n=3 biological independent samples. **j** Pathways downregulated following VAX2 knockdown in MDA468 cells include those involved in mesenchymal development, ECM organization, and collagen synthesis. Statistical analysis was performed using a two-sided Student's t-test. *** *p* < 0.001.


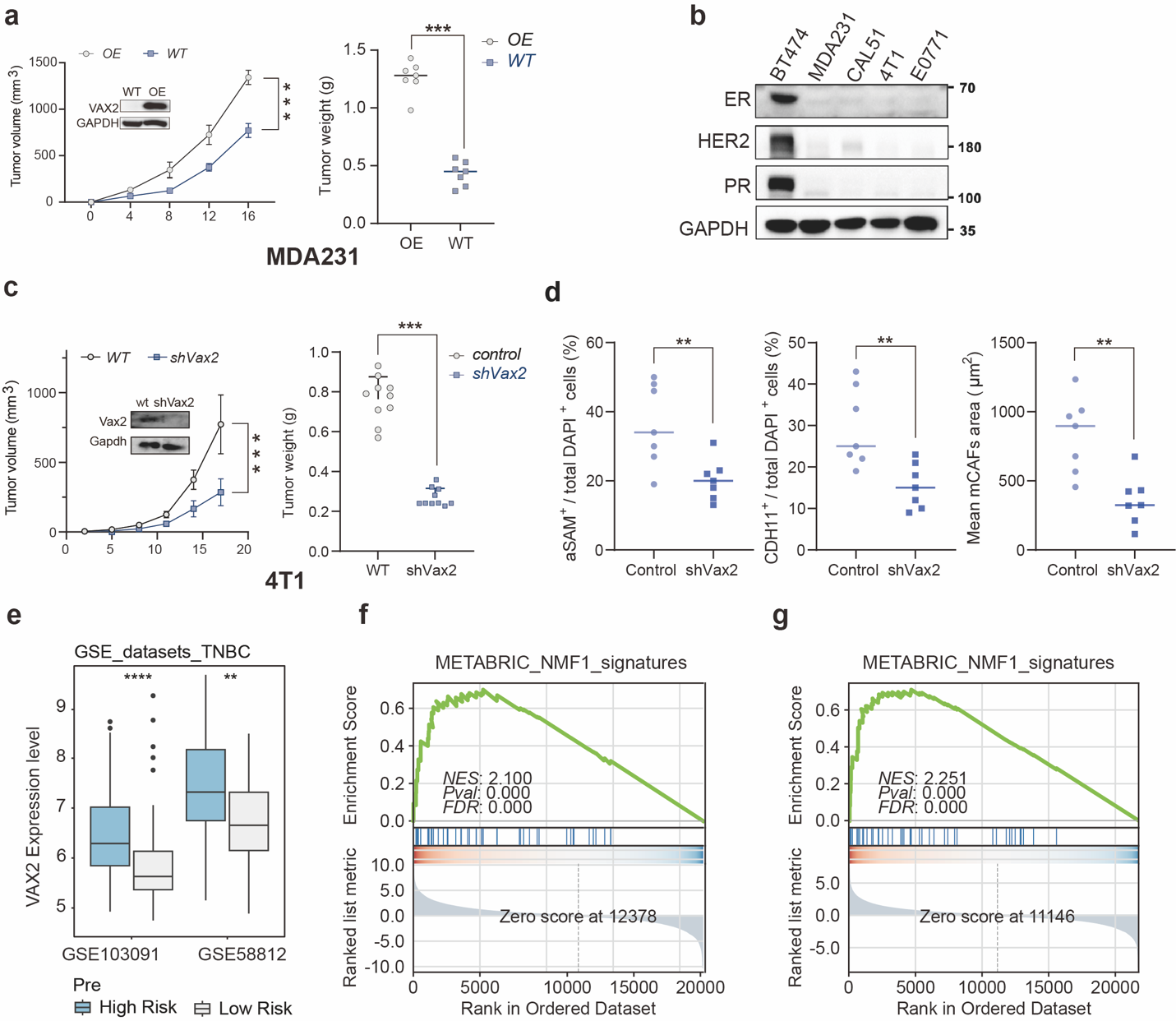


**Supplementary Fig. 7 Tumor-associated biological functions of VAX2 as a key master regulator in the TNBC mesenchymal subtype. a** Tumor weight measurements and growth curves of VAX2-overexpressing and wild-type MDA231 tumors transplanted into BALB/c nude mice. **b** Western blotting showed the expression of ER, PR and HER2 in E0771 and 4T1 cell line. **c** Tumor weight measurements and growth curves of VAX2-knockdown and wild-type 4T1 tumors transplanted into BALB/c mice. **d** Quantification and statistical analysis of cells positive for different molecules in multiplex immunofluorescence assays. **e** Boxplot showing the expression of VAX2 in the predicted mesenchymal and non-mesenchymal subtypes in the GSE103091-TNBC and GSE58812-TNBC cohorts. **f-g** In the GSE103091-TNBC (**f**) and GSE58812-TNBC (**g**) cohorts, GSEA revealed significant enrichment of mesenchymal subtype characteristics in predicted mesenchymal subtypes compared to non-mesenchymal subtypes. Statistical analysis of tumor growth curves was conducted using a two-way ANOVA; Statistical analysis of tumor weight measurements and immunofluorescence assays was conducted using a two-sided Student's t-test. ** *p* < 0.01, *** *p* < 0.001.
